# K-homology Nuclear Ribonucleoproteins Regulate Floral Organ Identity and Determinacy in Arabidopsis

**DOI:** 10.1371/journal.pgen.1004983

**Published:** 2015-02-06

**Authors:** Encarnación Rodríguez-Cazorla, Juan José Ripoll, Alfonso Andújar, Lindsay J. Bailey, Antonio Martínez-Laborda, Martin F. Yanofsky, Antonio Vera

**Affiliations:** 1 Área de Genética, Universidad Miguel Hernández, Campus de Sant Joan d’Alacant, Sant Joan d’Alacant, Alicante, Spain; 2 Division of Biological Sciences, Section of Cell and Developmental Biology, University of California San Diego, La Jolla, California, United States of America; Peking University, CHINA

## Abstract

Post-transcriptional control is nowadays considered a main checking point for correct gene regulation during development, and RNA binding proteins actively participate in this process. *Arabidopsis thaliana FLOWERING LOCUS WITH KH DOMAINS* (*FLK*) and *PEPPER* (*PEP*) genes encode RNA-binding proteins that contain three K-homology (KH)-domain, the typical configuration of Poly(C)-binding ribonucleoproteins (PCBPs). We previously demonstrated that *FLK* and *PEP* interact to regulate *FLOWERING LOCUS C* (*FLC*), a central repressor of flowering time. Now we show that FLK and PEP also play an important role in the maintenance of the C-function during floral organ identity by post-transcriptionally regulating the MADS-box floral homeotic gene *AGAMOUS* (*AG*). Previous studies have indicated that the KH-domain containing protein HEN4, in concert with the CCCH-type RNA binding protein HUA1 and the RPR-type protein HUA2, facilitates maturation of the AG pre-mRNA. In this report we show that *FLK* and *PEP* genetically interact with *HEN4*, *HUA1*, and *HUA2*, and that the FLK and PEP proteins physically associate with HUA1 and HEN4. Taken together, these data suggest that HUA1, HEN4, PEP and FLK are components of the same post-transcriptional regulatory module that ensures normal processing of the AG pre-mRNA. Our data better delineates the roles of *PEP* in plant development and, for the first time, links *FLK* to a morphogenetic process.

## Author Summary

Unlike animals, angiosperms (flowering plants) lack a germline that is set-aside early in embryo development. Contrariwise, reproductive success relies on the formation of flowers during adult life, which provide the germ cells and the means for fertilization. Therefore, timing of flowering and flower organ morphogenesis are critical developmental operations that must be finely regulated and coordinated to complete reproduction. *Arabidopsis thaliana FLOWERING LOCUS WITH KH DOMAINS* (*FLK*) and *PEPPER* (*PEP*) encode two KH-domain RNA-binding proteins phylogenetically related to human proteins characterized by their high developmental versatility. FLK and PEP modulate the mRNA expression of the MADS-box gene *FLOWERING LOCUS C*, key in flowering control. In this work we have found that *FLK* and *PEP* also play a pivotal role in flower organogenesis by post-transcriptionally regulating the MADS-box floral organ identity gene *AGAMOUS* (*AG*). Interestingly, FLK and PEP physically interact with proteins involved in *AG* pre-mRNA processing to secure correct *AG* function in the floral meristem and flower. Taken together, our results reveal the existence of a post-transcriptional regulatory activity controlling key master genes for floral timing and flower morphogenesis, which might be instrumental for coordinating both developmental phases.

## Introduction

Development of multicellular organisms relies on exquisitely controlled transcriptional and post-transcriptional regulatory actions to govern gene expression and accurately respond to endogenous and environmental fluctuations. As exemplified in the reference plant *Arabidopsis thaliana* (Arabidopsis hereafter), reproductive success in angiosperms largely depends on two developmental events that initiate the reproductive phase: floral timing and flower morphogenesis. Upon flowering, the shoot apical meristem (SAM) transforms into an inflorescence meristem (IM) which will give rise to floral meristems (FMs) [1]. FM identity genes, such as *LEAFY* (*LFY*) [2] and *APETALA1* (*AP1*) [3], are crucial in activating the floral homeotic genes that specify identity of concentric whorls of organs in the Arabidopsis flower [1]. According to the ABC(E) model [4–6], the class A genes *AP1* and *AP2* specify sepals and, together with the B function genes *PISTILLATA* (*PI*) and *AP3*, contribute to petal identity. Co-expression of B-genes and the C-function gene *AGAMOUS* (*AG*) confer male stamen identity, while *AG* alone specifies female carpels, defining the pistil or gynoecium situated in the innermost whorl. The model also establishes mutual antagonism between A and C activities and requirement of the E activity, represented by the redundant *SEPALLATA* function [4–9]. With the exception of *AP2* (an AP2/EREBP) [10,11], all floral homeotic genes encode type II MADS-box transcription factors, a lineage comprising central regulators in most aspects of plant development [9,12,13].

In addition to floral organ identity, *AG* plays a crucial role in FM determinacy by repressing the homeobox stem-cell-identity gene *WUSCHEL* (*WUS*) [14,15]. *WUS* and *LFY* activate *AG*, which in turn, represses *WUS* both directly and through the activation of the transcriptional repressor *KNUCKLES* (*KNU*) [16], resulting in consumption of the stem cell niche [16–21]. Otherwise, continuing cell proliferation leads to an indeterminate pattern of alternating whorls of sepals and petals, as described in strong *ag* mutants [22].

Whereas transcriptional control of gene expression is key to development, it is nowadays widely accepted that post-transcriptional operations are crucial to secure proper gene regulation. For example, mounting evidence indicates that mRNA processing steps, such as splicing and polyadenylation, usually proceed co-transcriptionally in a tightly coordinated manner to ensure correct gene activity [23–25]. RNA-binding proteins from multifunctional ribonucleoprotein (RNP) complexes coat nascent transcripts to regulate different aspects of mRNA synthesis, affecting thus, the final levels of gene expression [26, 27].

It has been shown that, in addition to its transcriptional control, post-transcriptional regulation is essential to secure correct *AG* function during flower development, in particular *AG* intron 2 processing [28]. So far three Arabidopsis RNA-binding proteins (RNPs) were found to facilitate this process: HUA1, a nuclear CCCH-type zinc-finger protein [29], the RPR-domain (Regulation of nuclear pre-mRNA) protein HUA2 [30], and HUA ENHANCER 4 (HEN4), containing 5 K-homology (KH) domains and one of the few KH proteins functionally characterized in Arabidopsis [31,28]. Interestingly, *hua1 hua2 hen4* triple mutants displayed stamen and carpel homeotic transformations, and loss of flower determinacy as a result of the reduced levels of mature *AG* mRNA. The fact that HUA1 binds to the *AG* pre-mRNA and physically associates with HEN4, suggests that both proteins belong to the same RNP regulatory complex [28].

Named after the human heterogeneous nuclear ribonucleoprotein K (hnRNP K) [32], the KH domain is an ancient RNA-binding module present in proteins whose disruption causes important developmental alterations in animals, including human syndromes as fragile-X [33,34], metastasis and cancer progression [35]. The hnRNP K is also representative of the remarkably versatile poly(C)-binding proteins (PCBP), characterized by a stereotypical triple-KH-domain configuration. PCBPs play roles in multiple developmental processes in animal systems, from erythropoiesis to neuronal differentiation [36–40]. The KH domain also provides a structural basis for protein-protein interactions, which most likely contributes to the multifunctionality of PCBPs [36,41].

In contrast, very little is known about plant PCBP-type hnRNPs and their relevance to plant development or morphogenesis is largely unexplored. So far, only two canonical PCBP-type hnRNP encoding genes, *FLOWERING LOCUS WITH KH DOMAINS* (*FLK*) [42,43] and *PEPPER* (*PEP*) [44], have been characterized in Arabidopsis to some extent. *FLK* promotes flowering in the autonomous pathway by negatively regulating the MADS-box floral repressor *FLOWERING LOCUS C* (*FLC*) [42,43,45]. *PEP* was originally described to interact with element(s) of the *WUS* pathway [44] and more recently we found that *PEP* is a positive regulator of *FLC* activity, hence antagonizing with *FLK* [46]. In line with this, the late flowering phenotype of *flk* plants (due to elevated levels of *FLC*) is rescued in the *flk pep* background [46]. However, in spite of the fact that *PEP* is expressed in FM and developing flowers, *pep*, *flk* or *pep flk* double mutants lack conspicuous floral defects, probably reflecting the compensation by overlapping activities [43,44,46].

In this work, we have functionally investigated the magnitude of *PEP* and *FLK* roles in flower patterning. Our genetic and molecular analyses place *PEP* as a positive regulator of the floral C-function by facilitating *AG* pre-mRNA processing and preventing premature polyadenylation in the large second intron. Here we also show that *FLK* also contributes to maintain the C-function. Furthermore, we provide evidence that PEP and FLK interact with the previously identified *AG* mRNA processing factors HUA1 and HEN4, strongly suggesting that all these proteins likely work together as components of a common post-transcriptional regulatory activity. Identifying *PEP* and *FLK* as new regulators of *AG*, broadens the scope of the developmental functions played by plant PCBPs, as they impinge upon the control of master regulatory genes, in this case *AG*, central during reproductive development.

## Results

### Genetic interactions with *HEN4*, *HUA1* and *HUA2* uncover the contribution of *PEP* to maintain the floral C-function

As mentioned above, *PEP* is expressed in FM and developing flowers, but *pep* flowers are largely normal. Thus, to test whether the role of *PEP* in floral patterning was masked by redundant gene activities, we combined the null *pep-4* allele [44] (*pep* hereafter) with mutations in *HEN4*, *HUA1* and *HUA2*, genes that encode post-transcriptional regulatory proteins [28].

**Fig 1:**
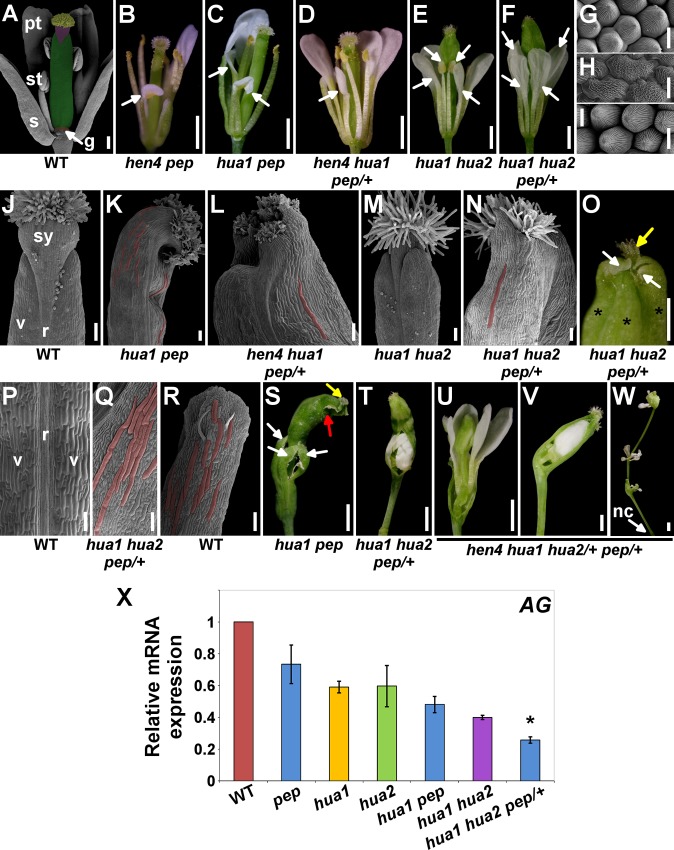
*PEP* regulates flower reproductive organ identity and determinacy. A) Scanning Electron Microscopy micrograph (SEM) of a post-anthesis wild-type flower. The different parts of the pistil have been artificially colored: stigma (yellow), style (purple), ovary (green), gynophore (g, orange). pt, petal; st, stamen; s, sepal. B-F) Post-anthesis flowers of different mutant backgrounds. Some outer organs were removed to better show petaloid stamens in the third whorl (arrows). G-I) SEM micrographs. Petal (G) and anther (I) adaxial surface in wild-type. Adaxial side in third whorl organs in *hua1 hua2 pep/+* flowers (I). J) SEM apical portion of wild-type pistil. K-N) Top portion (SEM micrographs) of gynoecia/fruits from different mutant combinations. O) *hua1 hua2 pep/+* pistils developed extra valves (asterisks) topped by white tissue that resembled that of sepal tips (white arrows). No style and very rudimentary stigmatic tissue were observed (yellow arrow). P) Medial view (SEM micrograph) of stage 17 wild-type fruit (according to [106]). Q) Abaxial surface of *hua1 hua2 pep/+* gynoecia. R) Abaxial side of a wild-type sepal. S) A *hua1 pep* flower after outer organ abscission. Sepaloid carpels (white arrows) formed the fourth whorl that developed on a long gynophore. Similar structures were seen developing inside the sepaloid gynoecia which also contained rudiments of stigmatic tissue (yellow arrow) and further additional floral organs (red arrow). T) *hua1 hua2 pep/+* fourth whorl. U-W) *hen4 hua1 hua2/+ pep/+* flowers. Fourth whorl organs were removed to observe inner flowers (U, V). X) mRNA expression levels of *AG* in the wild type (WT) and diverse *hua-pep* mutant backgrounds, monitored by quantitative RT-PCR (qPCR). Error bars, standard deviation (SD). Asterisks indicate statistically significant differences from *hua1 hua2* plants (*P < 0.05). On panels (K, L, N, Q) and (R) some giant cells appear false-colored. Scale bars: 1 mm (B, C, D, E, F, S, T, U, V, W), 500 μm (O), 200 μm (A), 100 μm (J, K, L, M, N, P, Q, R) and 10 μm (G, H, I). nc, nectaries; r, replum; sy, style; v, valve.

*HEN4* is a KH paralog relatively distant to *PEP* [31]. Unlike *hen4-2* (*hen4* hereafter) and *pep* single mutants (S1A Fig.) [28,44], ∼10% of *hen4 pep* flowers exhibited petaloid stamens (Fig. 1A, 1B, and S2B-S2D Fig.). Similarly, *hua1-1* mutants (*hua1* hereafter) appeared normal (S1B Fig.) [30], but *hua1 pep* double mutants displayed abundant petaloid transformations in the third whorl (40% of the flowers examined; Fig. 1C). We could not obtain *hen4 hua1 pep* triple homozygous mutants implying that *PEP* becomes essential in the *hen4 hua1* background. This was noteworthy since *hen4 hua1* double mutants flowers look wild-type [28]. Strikingly, introducing only one *pep* allele into *hen4 hua1* plants (*hen4 hua1 pep/+*) led to conspicuous floral alterations including petaloid stamens in all flowers (Fig. 1D).

Loss of *HUA2* does not cause any obvious floral phenotype [30] and, although *HUA2* and *PEP* interact during floral timing, *hua2-4 pep* flowers are normal [46]. However, this might not be surprising as our data indicate that the *hua2-4* allele is leaky (S3 Fig.). We therefore used the null *hua2-7* allele (*hua2* hereafter, unless it is specified otherwise). Double mutants *hua1 hua2* showed a variety of flower defects, including stamen-to-petal transformations (Fig. 1E and S1 Table), as reported for *hua1 hua2-1* [30]. Unexpectedly, we were unable to isolate *hua2 pep* or *hua1 hua2 pep* individuals and only *hua1 hua2 pep/+* plants were identified among the progeny. This background was sterile and showed a significant enhancement of the *hua1 hua2* floral phenotype, including stronger petaloid transformations (Fig. 1E-I and S1 Table).

### Dramatic alterations of fruit morphogenesis in *pep*, *hua* and *hen* mutant combinations

The fruit derives from the fertilized gynoecium carpels, whose formation, in turn, almost entirely depends on C-function [7,47]. We therefore decided to use carpel and fruit development as readout of how *pep*, *hua*, and *hen* mutant combinations affect C-function.

Although fruits in some of the mutant backgrounds were slightly shorter but normal looking (100% in *hen4 pep*, 20–60% in *hua1 pep*), we detected pistils with very distorted development, such as unfused carpels, and reduced style and stigma (S1D Fig.). In certain combinations, the apical portion of carpels was pointed with areas of white or pale green tissue conformed by smaller fringe cells as those in the apex of wild-type sepals (Fig. 1K, 1L, 1N, 1O and S2F, S2G Fig.).

The *hua1 hua2* double mutant presented shorter pistils broadened at the tip [30] (Fig. 1M and S1D Fig.). However, *hua1 hua2 pep/+* pistils were on average much shorter and crumpled (S1D Fig.). Indeed, close inspection of severely affected gynoecia in *hua1 hua2 pep/+* by scanning electron microscopy (SEM) revealed that the carpel epidermis, rather than the wild-type characteristic vertical files of smooth cells (Fig. 1P), showed a wide range of epidermal cell sizes with epicuticular wax crenulations, including sepal-like giant cells [48–50] (Fig. 1N, 1Q, 1R). These alterations are typical of carpel-to-sepal transformation and were also seen in additional *pep* mutant combinations (Fig. 1K, 1L and S2H-S2K Fig.).

We detected that a significant percentage of *hua1 pep* pistils (40%) developed supernumerary valves (S1 Fig. and S2L Fig.). This trait is typical of loss of meristem determinacy and it was further enhanced in *hua1 hua2 pep/+* (Fig. 1O and S1 Table). Terminal *hua1 pep* flowers, and at least a quarter of the *hua1 hua2 pep/+* flowers exhibited conspicuously long gynophores and gynoecia that, strikingly, contained additional flowers inside. These basically consisted of petals and sepaloid gynoecia recapitulating the sepaloid features seen in the fourth whorl (Fig. 1S, 1T and S2M, S2O Fig. and see below). This phenotype, never observed in *hua1 hua2* flowers (S1 Table), was reminiscent of that of *ag* mutants and also resembled the loss of *HEN4* in the *hua1 hua2* background [28]. In *hen4 hua1 pep/+* a significant fraction of flowers (25%) contained supernumerary sepaloid valves (S2N Fig.), reflecting certain loss of determinacy in this genotype.

Overall, these results indicate that *PEP*, in collaboration with *HUA* and *HEN* genes, act as a positive regulator of the floral C-activity to, therefore, secure the downstream developmental programs depending on this function, such as fruit development.

The mutant combinations described above exhibit very similar developmental defects. Moreover, gene dosage effects in *hua1 hen4 pep/+* and *hua1 hua2 pep/+* plants illustrate the sensitivity of such backgrounds to *PEP* activity. These findings strongly suggest that PEP shares redundant developmental functions with HUA1, HUA2 and HEN4 despite their protein structural disparity. Accordingly, *hen4 hua1 hua2/+ pep/+* plants showed very dramatic floral alterations (Fig. 1U-W and S2P-S2R Fig.). Hence, these factors were tentatively included in a common gene activity abbreviated as *HUA-PEP* along this work.

### The lack of *HUA-PEP* activity causes sepaloid transformations in *ful* gynoecia

**Fig 2:**
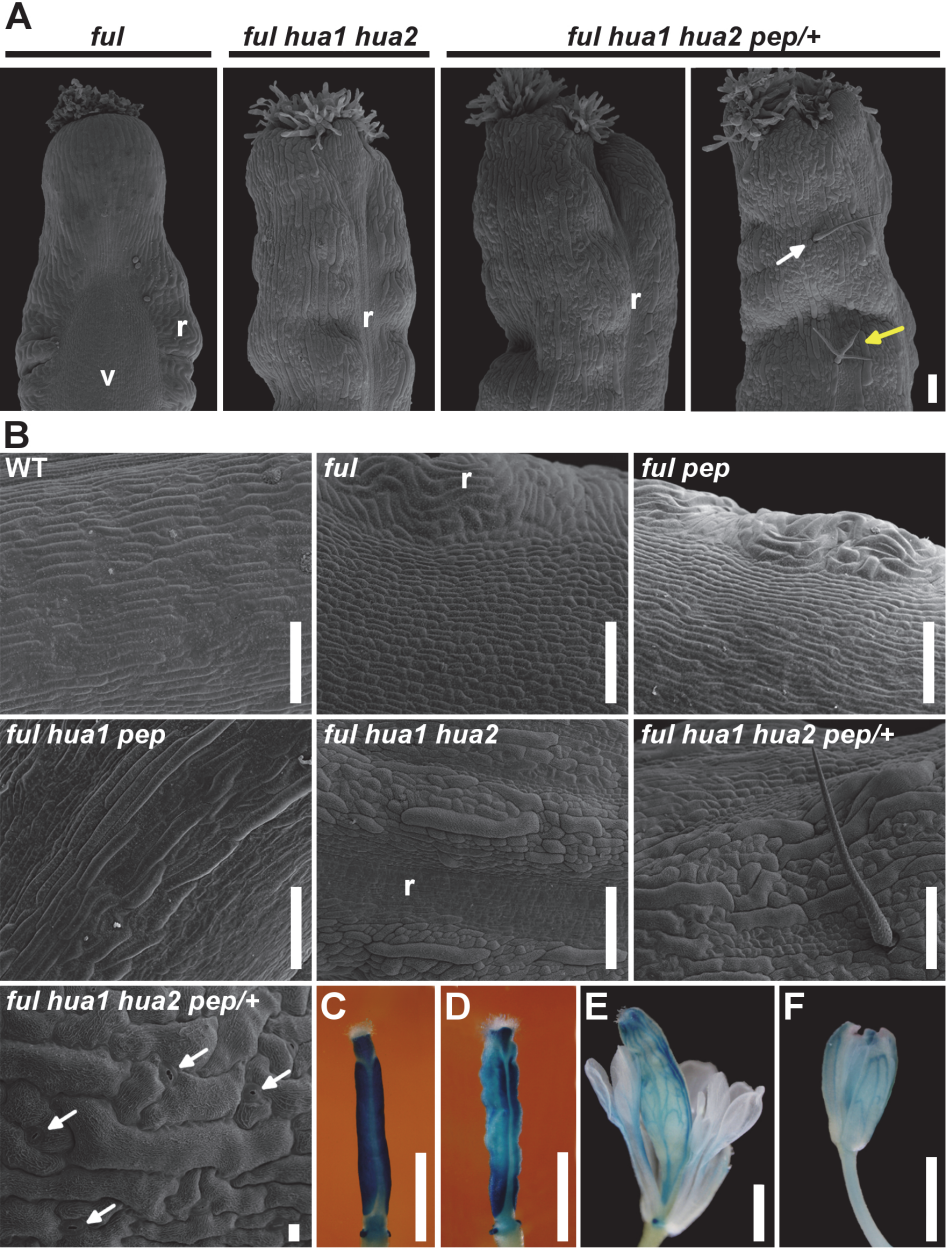
The loss of *HUA-PEP* activity is epistatic over the *ful* phenotype. A) SEM images of the top portion of a *ful* fruit. The typical long style and wide zig-zag replum were suppressed in *ful hua1 hua2* and *ful hua1 hua2 pep/+* pistils, and sepaloid giant cells were observed on the valve surface. Simple or branched trichomes (white and yellow arrows, respectively) were occasionally observed on the surface of *ful hua1 hua2 pep/+* pistils. B) SEM images of the abaxial ovary surface in wild-type (WT) and different mutant backgrounds. Observe interspersed stomata (arrows) in a *ful hua1 hua2 pep/+* panel. C-F) *GUS* reporter whole-mount staining (*ful-1*) in *ful* (C), *ful hua1 hua2* (D), *ful hua1 hua2 pep/+* (E) pistils and wild-type sepal (F). Observe long gynophores and full petaloid conversion of stamens in (E). Scale bars: 100 μm (A, B), except 10 μm in the last B panel (*ful hua1 hua2 pep/+* genotype), and 1 mm (C-F). r, replum; v, valve.

The MADS-box regulatory gene *FRUITFULL* (*FUL*) [51] is crucial for valve formation during ovary patterning, and it does so, in part, by preventing valves from adopting valve margin identity through the negative regulation of valve margin identity genes [52–56]. Upon fertilization, *ful* lignified valve cells remain small, arresting stomata development and silique growth. However, replum cells develop normally leading to a characteristic zig-zag configuration of this tissue in *ful* fruits [51] (Fig. 2A, 2B and S4A Fig.). Additionally, *ful* siliques show elongated styles [57] (Fig. 2A and S4A, S4K Fig.).

To get more insights into the role of *HUA-PEP* activity during pistil development, we decided to characterize the behavior of *hua-pep* activity mutants in the *ful* background.

The *ful-1 hua1* fruit was virtually identical to that of *ful-1* plants [51] (*ful* hereafter; S4A Fig.). In contrast, *ful pep*, *ful hua2* and *ful hua1 pep* siliques were progressively longer and showed shorter styles (S4A Fig.). In such backgrounds, valve epidermal cells were elongated and streaked, along with interspersed stomata. These phenotypes indicated that valves took onto sepaloid identity (Fig. 2B and S4B Fig.). In *ful hua1 hua2*, gynoecia were smaller and replum cells remained small as in wild-type unpollinated pistils [58], abolishing the characteristic zig-zag shape (Fig. 2A, 2B and S4A Fig.). Fertility in *ful hua1 hua2* plants was severely reduced.

*ful hua1 hua2 pep/+* plants were phenotypically identical to *hua1 hua2 pep/+*. In such combinations, we also found new floral organs developing inside swollen gynoecia that were often seating on long gynophores (Fig. 2E and S4A, S4E, S4I, S4L, S4M Fig.).

The glucuronidase (GUS) reporter harbored by the *ful-1* transposon reflects the native expression pattern of *FUL* [51]. Pistils of *ful* or wild-type-looking heterozygous *ful/+* plants displayed characteristic *GUS* activity in the valves, style and nectaries [51] (Fig. 2C and S4D, S4G Fig.). In *ful hua1 hua2*, strong *GUS* signal was detected in nectaries and apical territory preserving style identity, whereas valves presented a more irregular pattern (Fig. 2D and S4H Fig.). The GUS-staining pattern of *ful hua1 hua2 pep/+* in the fourth whorl organs had little resemblance to that of a gynoecium, except in nectaries and style vestiges, notably evoking *FUL* expression in the sepal vasculature [51] (Fig. 2E, 2F and S4E, S4F, S4I Fig.).

Next, we treated flowers with the lignin-specific dye phloroglucinol. Mature wild-type fruits showed preferential staining in the valve margin, whereas in *ful* mutants valves were ectopically lignified [52,53] (S4J, S4K Fig.). Nonetheless, in equivalent flowers from *ful hua1 hua2 pep/+* plants, lignification in the presumptive gynoecium was nearly restricted to branched red lines with striking resemblance to lignified sepal vasculature (S4L-S4N Fig.). Altogether, genetic and histochemical analyses indicate that the *HUA-PEP* gene activity is required to prevent gynoecium tissues from adopting sepaloid fate independently of their original identity (valve, valve margin), highlighting the role played by *PEP* to preserve carpel identity.

### *PEP* is a positive regulator of *AG* functional mRNA levels

To determine whether *PEP* impinges upon *AG* regulation and therefore C-function, we measured mRNA levels from wild-type and mutant flower buds by quantitative PCR (qPCR). In consonance with the phenotypes described above, relative expression of *AG* decreased significantly in *hua1 pep* and *hua1 hua2* double mutants, and reduced even further in *hua1 hua2 pep/+* plants (Fig. 1X).

To investigate whether somehow *PEP* (and *HUA*) control A and B function, we measured the transcript levels of the homeotic A- and B-class genes *AP1* and *PI*, respectively. Results were inconclusive because, although expression of both genes declined moderately in some mutant strains (S5 Fig.), no morphological evidence of altered A- or B-floral functions was observed in any of the *hua-pep* mutant combinations examined. Therefore, our molecular and genetic data suggest that, in contrast to the C-function, it appears that *HUA-PEP* gene activity has little or no role in regulating A- and B-functions.

*AG* triggers several reproductive developmental programs in part by activating additional regulators that perform different subsets of its functions. For example, *SPOROCYTELESS* (*SPL*) stimulates stamen development, including organ identity [59–61], whereas the zinc-finger gene *KNU* cooperates with *AG* to repress *WUS* [19,21]. Consistently, *SPL* and *KNU* expression decreased markedly in *hua1 pep* and *hua1 hua2* double mutants, and *hua1 hua2 pep/+* plants (S6A, S6B Fig.). Accordingly, *KNU* gene expression monitored by a GUS-reporter construct was found to be less intense in *hua1 pep* developing flower organs, as compared to the wild type (S6C-S6H Fig.).

Interestingly, we found that the mutant phenotype of *hua1 pep* plants was completely rescued by increasing the dosage of *AG* gene with a genomic construct able to complement *ag* mutants [62] (S7 Fig.), thus reinforcing our hypothesis that *AG* functions depend on *HUA-PEP* activity genes. Collectively, these results might explain the organ identity and determinacy defects seen in *pep hen hua* combos and further support *PEP* as a positive regulator of *AG*.

### *PEP* prevents *AP1* expansion to inner whorls

**Fig 3:**
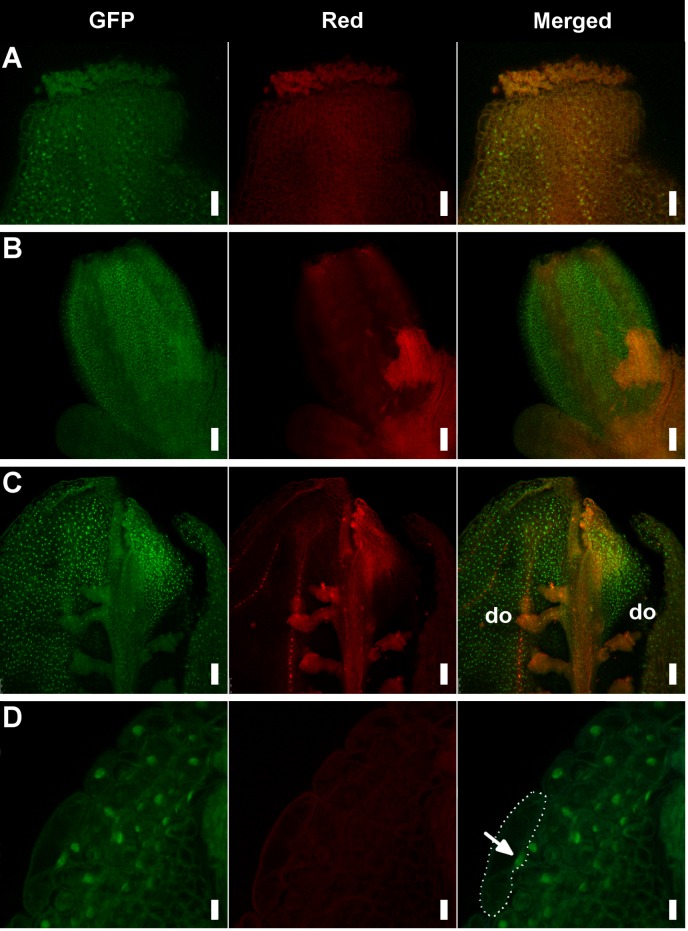
Detection of the AP1-GFP protein in *hua1 pep* gynoecia. A) Apical region of a mildly affected gynoecium with recognizable pistil morphology. Specific AP1-GFP signal is detected in some style cells. B) Fourth whorl organs of a pre-anthesis flower displaying a severe sepaloid phenotype. C) Adaxial (inner) view of a manually open pistil with severe sepaloid transformations, but containing some developing ovules (do). D) Detail of a fourth whorl organ from panel D showing nuclear-localized AP1-GFP. A cell has been outlined with a dotted line and the nucleus marked with an arrow. Scale bars: 25 μm (A), 50 μm (B and C) and 10 μm (D).

One of the functions of *AG* is to prevent *AP1* expression in the two inner whorls of organs where stamens and carpels normally form [8]. To examine the expression of *AP1* we used the genomic GFP (green fluorescent protein)-based reporter *gAP1*::*AP1-GFP*, that largely mirrors endogenous AP1 expression [62]. As expected, in the wild type AP1-GFP signal was detected in sepals but absent in pistils (S8 Fig.). However, a number of *hua1 pep* pistils showed AP1-GFP fluorescence (Fig. 3A-D). These results are coherent with earlier work showing *AP1* mRNA ectopic expression in inner whorls of *hua1 hua2* and *hua1 hua2 hen4* [28,30], and underscore the importance of *PEP* as a regulator of the C-function during flower organogenesis.

### *PEP* overexpression impairs flower morphogenesis

**Fig 4:**
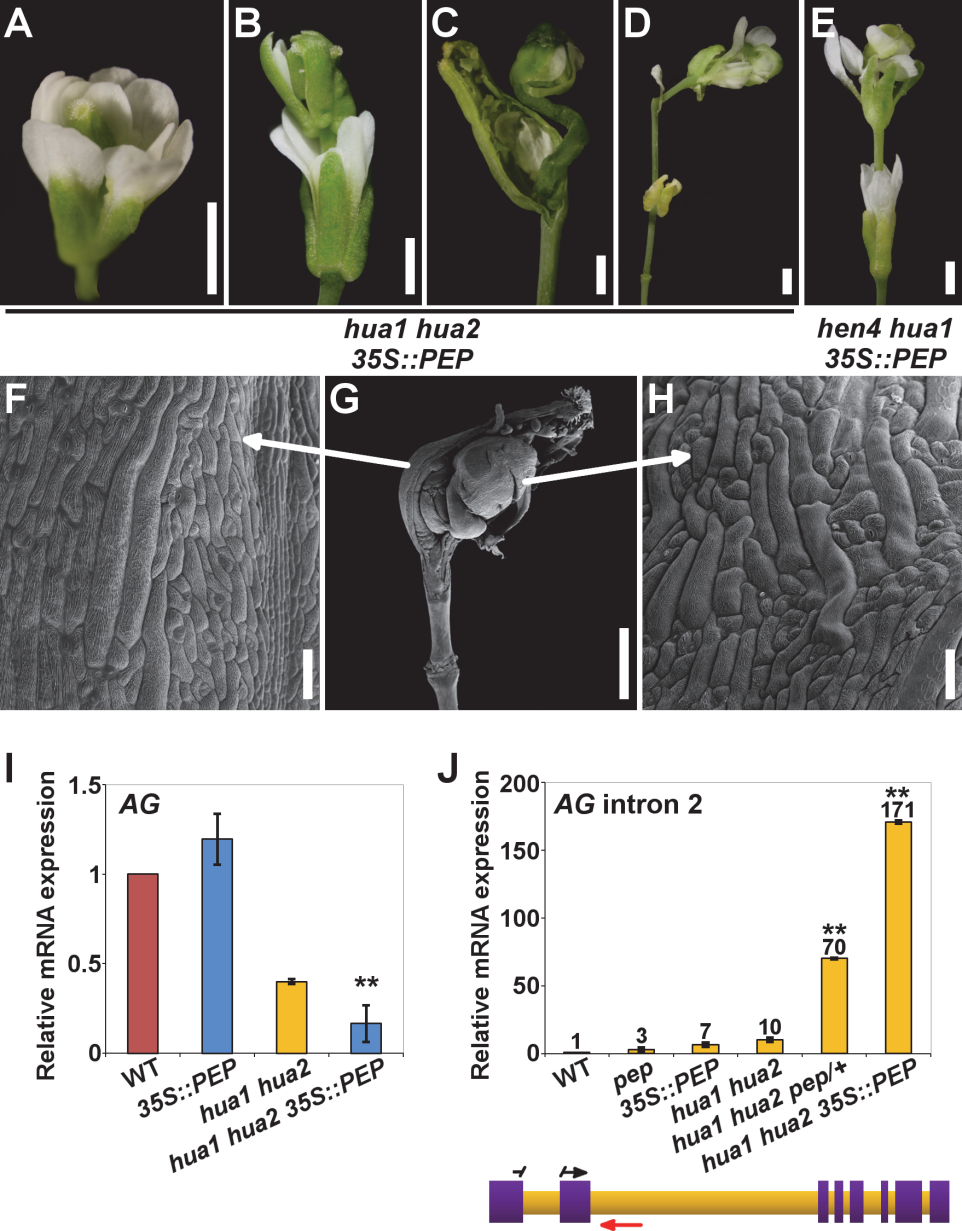
*PEP* overexpression impairs flower morphogenesis and *AG* pre-mRNA processing. A-D) *hua1 hua2 35S*::*PEP* flowers. E) *hen4 hua1 35S*::*PEP* flower. In both genotypes, loss of determinacy was frequent. All flowers displayed severely transformed petaloid stamens and sepaloid carpels. F-H) SEM micrograph of a *hua1 hua2 35S*::*PEP* flower (G), and close-up views of the fourth whorl organ abaxial surface (F) and inner additional whorl organ (H), respectively. Sepaloid traits were found in these gynoecia. I, J) Relative expression levels of *AG* mRNA (I) and *AG* transcripts including intron 2 sequences (J), in wild-type plants (WT) and diverse *hua-pep* mutant backgrounds, monitored by qPCR. In (J), a diagram of the *AG* gene is shown below. Purple boxes denote exons whereas intronic regions are colored in orange. Relative positions of forward (black arrow) and reverse (red arrow) primers are indicated. To increase annealing specificity, the forward primer sequence was split between exons 1 and 2. Error bars, SD. Asterisks indicate statistically significant differences from *hua1 hua2* plants (**P < 0.01). Scale bars are 1 mm (A-E, G) and 100 μm (F, H).

Our loss-of-function genetic analyses show that components of the *HUA-PEP* function are redundantly required for the floral C-function. So we asked whether *PEP* alone could compensate for the deficiency in members of this activity. To test this idea, a *35S*::*PEP* overexpressing construct [46] was introduced into the *hua1 hua2* background. Strikingly, *PEP* overexpression, instead of rescuing, dramatically enhanced the *hua1 hua2* mutant phenotypes. Homozygous *hua1 hua2 35S*::*PEP* flowers were sterile, and exhibited much stronger stamen-to-petal and carpel-to-sepal transformations than in *hua1 hua2*, as well as frequent severe indeterminacy defects, a trait never observed in *hua1 hua2* plants (Fig. 4A-D, 4F-H, S9A-S9E Fig. and S1 Table). In line with the strong phenotypes observed, the levels of *AG*, *KNU* and *SPL* mRNAs in *hua1 hua2 35S*::*PEP* plants were significantly lower than those of *hua1 hua2* mutants (Fig. 4I and S9K, S9L Fig.).

We ruled out any RNA silencing effect as *hua1 hua2 35S*::*PEP* plants showed much higher *PEP* mRNA levels than wild-type individuals (S10D Fig.). Rather, PEP protein overproduction might exceed a certain critical threshold, leading to the strong phenotypes observed. Consistent with this idea, hemizygous *hua1 hua2 35S*::*PEP/+* plants produced *PEP* mRNA levels higher than those of the wild type, yet much lower than in homozygous *hua1 hua2 35S*::*PEP* plants (S10D Fig.), and did not show the severe floral phenotypes of the latter, being indistinguishable from *hua1 hua2* individuals (S10E-J Fig. and S1 Table).

Although, *PEP* overexpression in *hen4*, *hua1* and *hua2* single mutant backgrounds did not result in noticeable morphological alterations (S10A-C Fig.), we speculated whether excess of PEP was critically detrimental in more compromised conditions. In line with this interpretation, *PEP* overexpression in the wild-type looking *hen4 hua1* plants [28] led to the same developmental abnormalities previously described for the strong deficient *hua-pep* backgrounds. A significant number of *hen4 hua1 35S*::*PEP* flowers (∼65%) displayed severe indeterminacy, closely resembling *ag* flowers (Fig. 4E and S9F-J Fig.). It is worth mentioning that this phenotype never occurred in *hen4 hua1 pep/+*, indicating that *PEP* gain-of-function has a stronger impact on floral determinacy in *hen4 hua1* than reducing *PEP* activity, similarly as described for *hua1 hua2* background (S1 Table).

### *PEP* secures correct *AG* function by facilitating pre-mRNA processing

Mutations in *HEN4*, *HUA1* and *HUA2* led to a gradual decrease of *AG* mRNA levels concomitant with the accumulation of aberrant transcripts incorrectly terminated at the large second intron [28]. To test whether *PEP* impacts on this process, we carried out qPCR assays using intronic primers situated near the exon2/intron2 junction (Fig. 4J and S2 Table). The relative abundance of a PCR product increased progressively in various *hua-pep* mutant strains, notably in *hua1 hua2 pep/+* and *hua1 hua2 35S*::*PEP* individuals, whereas it was barely detectable in the wild type (Fig. 4J). These values negatively correlated with the levels of correctly spliced *AG* transcript in the mutant backgrounds under study, and unambiguously indicated that altering levels of *PEP* has an important impact on the accumulation of these transcript species.

To examine transcript structure, polyadenylated RNA from *hua1 hua2 pep/+* and *hua1 hua2 35S*::*PEP* plants was subjected to 3’ RACE (Rapid Amplification of cDNA Ends). Several products were obtained corresponding to transcripts comprising correctly spliced exons 1 and 2 followed by a variable stretch of nucleotides of intron two (105–368 nt), after which premature cleavage and polyadenylation events took place (S11A, S11B Fig.). These transcripts miss the last 6 exons, lacking the ability to encode a functional AG polypeptide. In plants, three polyadenylation signals define the site of processing: the far upstream element (FUE), the near upstream element (NUE), and the cleavage element (CE) [63]. Inspection of such RACE products revealed the presence of FUE, NUE and CE elements properly situated, strongly suggesting their implication in the premature termination event [63] (S11A, S11B Fig.).

### *FLK*: an additional component of the *HUA-PEP* activity

*FLK* is expressed in all major organs, yet its loss of function did not cause any visible defect [42,43]. *FLK* interacts with *PEP* and *HUA2* during flowering time regulation [46] but its possible role in flower morphogenesis has not yet been studied.

To explore *FLK* activity during flower development and to determine whether *FLK* participates in the *HUA-PEP* function, the null *flk-2* mutant [43] (*flk* hereafter) was crossed to different *hua-pep* mutant combinations. *flk hen4* double mutant flowers were wild-type in appearance (S12A, S12B Fig.). Unlike *hua1 pep* (Fig. 1 and S1 and S2 Fig.), *flk hua1* and *flk pep* double mutant flowers also looked essentially normal (S12C Fig.) [46]. In contrast, *flk pep hua1/+* plants showed some aberrant gynoecia, and petaloid stamens (S12D-F Fig.). Interestingly, stamen identity in the *flk pep* background, therefore, is sensitive to *HUA1* gene-dosage since this trait is never observed in *pep hua1/+*, nor in *flk pep* flowers.

**Fig 5:**
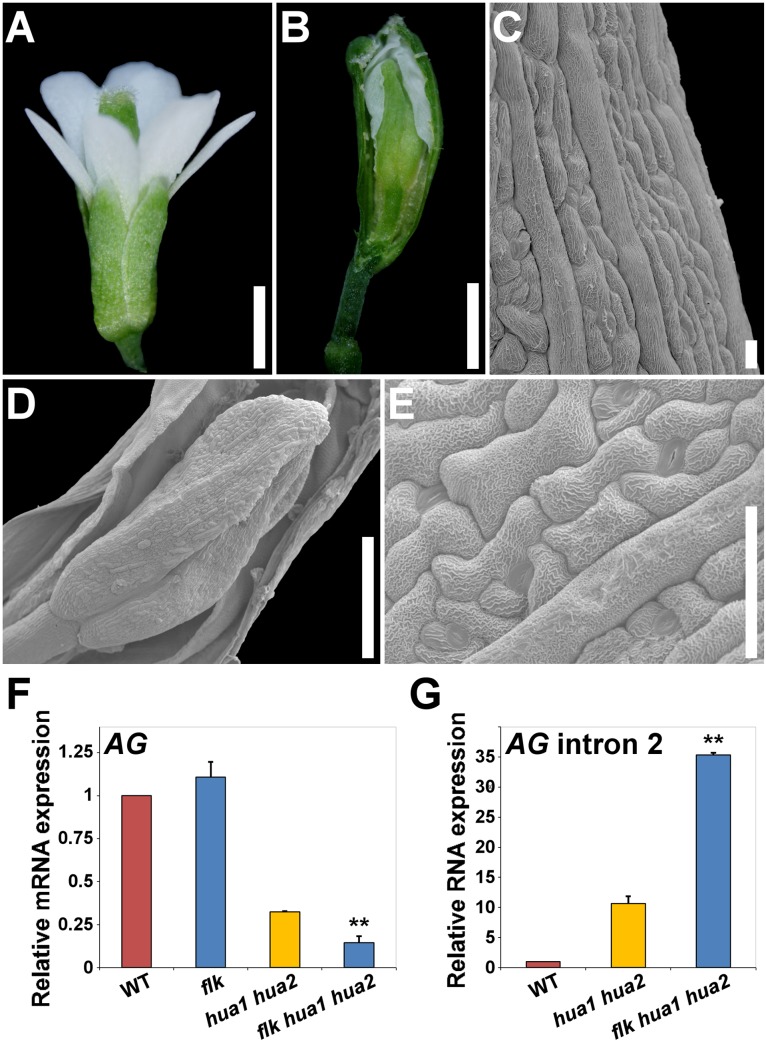
Loss of *FLK* dramatically enhances the floral phenotypes of *hua1 hua2* plants. A) *flk hua1 hua2* flower with all stamens converted into petals. B) Gynoecium with a long gynophore. A sepaloid valve was manually removed to better observe a new flower developing inside. C) SEM image of a sepaloid carpel with giant cells and epicuticular wax ridges. D) SEM magnification of the inner flower shown in (B). E) Close-up view of the sepaloid organ shown in (D). F, G) qPCR relative expression levels of *AG* mRNA (F), and *AG* transcripts including intron 2 sequences (G) in wild type (WT) and mutant backgrounds. Error bars, SD. Asterisks indicate statistically significant differences from *hua1 hua2* plants (**P < 0.01). Scale bars: 1 mm (A, B), 20 μm (C) 500 μm (D) and 50 μm (E).

Next, the *flk* mutant was crossed to *hua1 hua2* plants, a sensitized background repeatedly used to uncover gene activities involved in flower organ identity and determinacy [28,64–66, this work]. The resulting *flk hua1 hua2* triple mutants were easily identified because of their conspicuous flower defects. *flk hua1 hua2* flowers had two sets of petals and were “stamenless” (Fig. 5A and S12G Fig.), thus lacking fertilization and fruit set. Besides, *flk hua1 hua2* gynoecium development was severely distorted with obvious sepaloid attributes (Fig. 5B, 5C). Nevertheless, the most defining feature was again the occurrence of indeterminate flowers (>50%) (Fig. 5B, 5D, 5E and S12H Fig.). As indicated above, *hua1 hua2* flowers never show this severe developmental alteration, underscoring the contribution of the *flk* mutation to debilitate the floral C-function.

Our qPCR gene expression data backed up the hypothesis of *FLK* as part of the *HUA-PEP* activity. In *flk* the expression levels of *AG*, *KNU* and *SPL* remained unaltered when compared to those of the wild type, whereas in *flk hua1 hua2* significantly dropped, being even lower than in *hua1 hua2* individuals (Fig. 5F and S12I Fig.). This result substantiates the floral defects detected. Conversely, levels of *AG* transcripts containing intron 2 sequences increased in *flk hua1 hua2* (Fig. 5G), suggesting an influence of *FLK* on *AG* post-transcriptional regulation. Indeed, we performed 3’ RACE assays for RNA from *flk hua1 hua2* and identified new aberrant transcripts indicating premature cleavage and polyadenylation within the large intron 2. As described above, polyadenylation signals were found around the presumptive maturation site (S11C Fig.). Altogether, these results strongly support *FLK* as an additional component of the *HUA-PEP* activity.

### The components of the *HUA-PEP* activity physically associate

**Fig 6:**
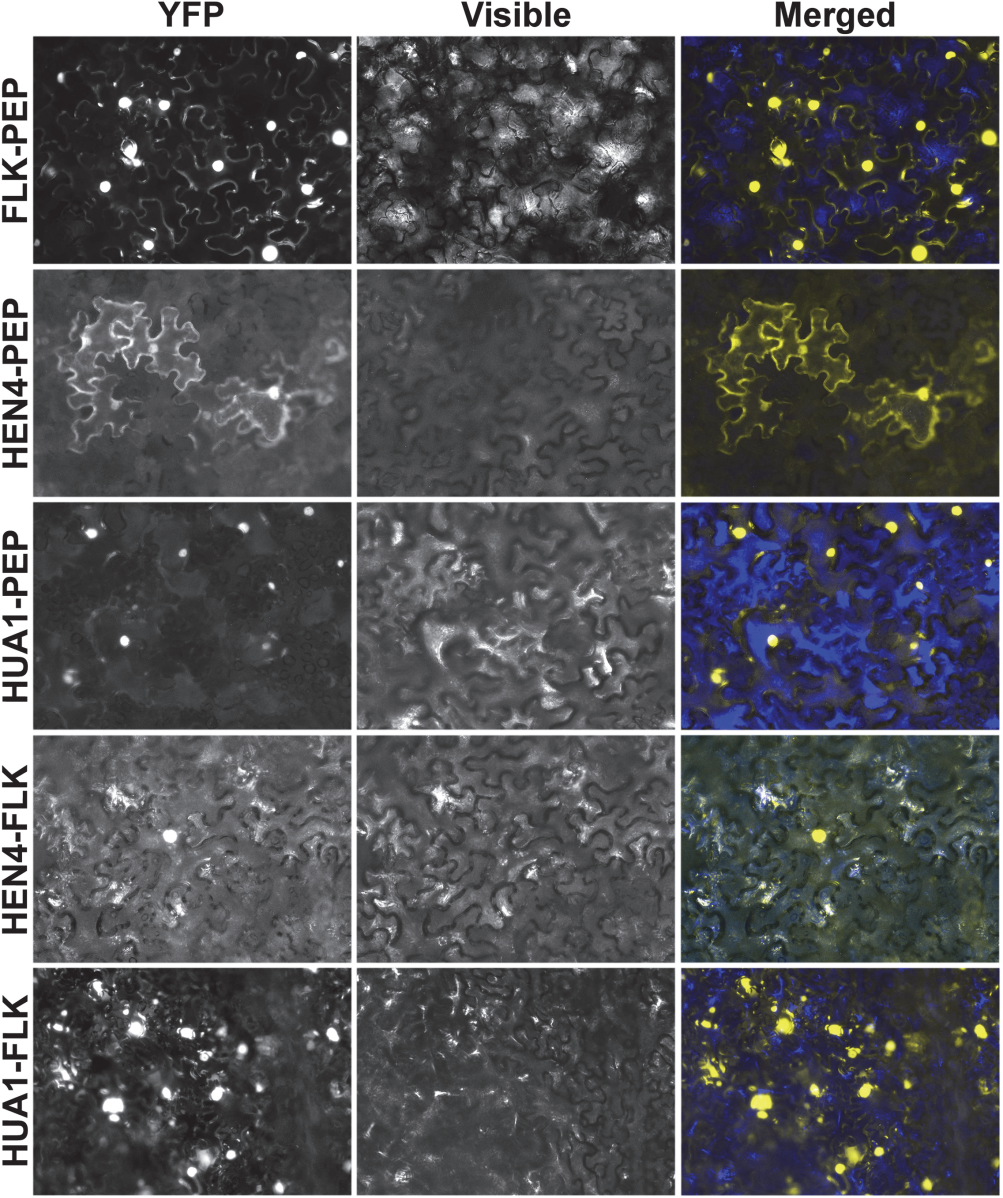
The hnRNPs PEP and FLK physically interact with HUA1 and HEN4. BiFC visualization of protein dimerization (yellow fluorescence) in *Nicotiana benthamiana* leaf cells agroinfiltrated with plasmids encoding fusion proteins. In each test, the first protein was fused to the C-terminal fragment of the YFP (YFPct), and the second protein to the N-terminal portion (YFPnt), respectively (see Materials and Methods section).

As mentioned in the introduction, RNA binding proteins participate in multimeric RNP complexes to perform their regulatory functions [36,41]. Our genetic and expression analyses indicated that genes of the *HUA-PEP* activity act in concert during floral organogenesis, which makes reasonable their interplay at the protein level. Nuclear localization of their products has been demonstrated [28,42,43,46,67] and, importantly, physical interaction between HEN4 and HUA1 has been already established [28]. Moreover, HEN4 was also computationally predicted to interact with PEP and FLK [68]. We therefore, conducted *in vivo* bimolecular fluorescence complementation (BiFC) assays in tobacco leaves using PEP, FLK, HEN4 and HUA1. Reconstituted yellow fluorescent protein (YFP) was detected in leaf cell nuclei when FLK-PEP, HEN4-PEP and HUA1-PEP interactions were assayed, respectively (Fig. 6 and S13 Fig.). Similarly, robust nuclear interaction was seen when FLK was tested against HUA1 and HEN4 (Fig. 6 and S13 Fig.). The HUA1-HEN4 BiFC interaction was used as a positive control (S13 Fig.). All associations were tested in both directions, thus endorsing specificity of the interactions (Fig. 6 and S13 Fig.).

We were also able to confirm *in vivo* protein homodimerization of PEP and FLK in our assays, corroborating the publicly available *in silico* data [68] (S13 Fig.). Homodimer formation was also seen in HUA1 BiFC experiments (S13 Fig.). These associations were further verified in yeast-two-hybrid assays (Y2H; S14 Fig.).

In a subset of our BiFC assays we detected, in addition to clear signal in the nuclei, specific cytoplasmic fluorescence (Fig. 6 and S13 Fig.). KH-domain containing proteins, particularly PCBPs, are known to participate in numerous RNA processing events in the nucleus and in the cytoplasm (RNA transport, stability, translation) [36–38]. Therefore, this extranuclear signal might reflect additional regulatory roles for PEP and FLK in this cell compartment.

Taken together, these results strongly suggest that PEP, FLK, HUA1 and HEN4 proteins physically associate likely reflecting their participation in common multimeric complexes involved in pre-mRNA processing. Additionally, these data further reinforce the assumption of *FLK* as a new partner of the *HUA-PEP* activity.

## Discussion

*PEP* and *FLK* were previously identified to control flowering time through regulation of the *FLC* gene [46,42]. Now, our analyses show that *PEP* and *FLK* also play a key role in the specification of flower organ identity as components of the post-transcriptional machinery that ensures normal processing of the *AG* pre-mRNA. Genetic, functional and molecular interactions with additional RNA-binding proteins previously established as *AG* regulators [28] led us to define *HUA-PEP* as a common gene activity comprising *HUA1*, *HUA2*, *HEN4*, *PEP* and *FLK*.

### *PEP* is a positive regulator of C-function activity during flower morphogenesis

We have demonstrated that *PEP* is functionally linked to the *AG* pre-mRNA processing pathway. Whereas *hua1*, *hua2* and *hen4* single mutants are phenotypically wild-type [28, this work], when these same mutants were combined with *pep*, we observed developmental abnormalities consistent with reduced C-function activity. Moreover, *hua1 hua2* double mutant flower defects [30] were dramatically enhanced when combined by plants that were heterozygous for a mutation in *PEP* (*hua1 hua2 pep/+*), illustrating dosage-effects among *HUA-PEP* genes as previously reported for *HUA1*, *HUA2* and *HEN4* [28]. The intensity of these floral phenotypes correlated with a reduction in *AG* mRNA levels. As a result, the A-function gene *AP1*, which is normally expressed in whorls 1 and 2, was ectopically expressed in the inner whorls of *hua1 pep* flowers, consistent with a compromised C-function and the A-C antagonism [4,8]. The sepaloid transformations seen in gynoecium tissues when *HUA-PEP* genes were mutated in the *ful* background provided further evidence for the critical contribution of *PEP* to carpel identity.

Loss of *PEP* contributed to reduce the floral C-function activity. Surprisingly, *PEP* overexpression in *hua1 hua2* and *hua1 hen4* also caused a dramatic enhancement of flower mutant phenotypes. Although this might seem unexpected, there are many examples in which loss- and gain-of-function result in the same phenotypical alterations. Loss and overexpression of *bancal*, encoding a *Drosophila* homologue of vertebrate hnRNP K, generates appendage developmental defects [69]. In *Xenopus* embryos, both reduction and overexpression of the KH gene *Mex3b*, involved in neural plate formation, led to downregulation of target genes [70]. In Arabidopsis, increasing or reducing the expression of kinase-encoding genes *FAB1A/B* elicits the same pleiotropic alterations, which are attributed to perturbations in the protein complexes in which they participate [71].

However, *PEP* overexpression in wild-type or single *hua-pep* mutant backgrounds rendered normal flowers, suggesting certain buffering capacity against PEP excess. Nevertheless, simultaneous inactivation of various HUA-PEP components (*hua1 hua2* or *hen4 hua1*) when *PEP* is overexpressed might aggravate a detrimental excess of PEP, by likely disrupting protein stoichiometric equilibria [72]. In line with this hypothesis is the fact that hemizygous *hua1 hua2 35S*::*PEP/+* plants, expressing higher levels of *PEP* than the wild type but much less than homozygous *hua1 hua2 35S*::*PEP* plants, do not differ from *hua1 hua2* double mutants.

### *FLK* is a member of the *HUA-PEP* gene activity

Our analyses have also uncovered a role for *FLK* in plant morphogenesis. *FLK* participates in the *HUA-PEP* activity during C-function maintenance. The genetic interaction between *flk*, *pep*, *hua1* and *hua2*, the phenotypic similarities between *flk hua1 hua2* (Fig. 5) and *hen4 hua1 hua2* [28], the gene expression analyses, as well as FLK physical associations, firmly support this conclusion.

*FLK* represses *FLC* and thus promotes flowering whereas *PEP* and *HUA2* are *FLC* activators [42,43,46,73,74]. During flower morphogenesis, however, *FLK* and *PEP* promote flower morphogenesis through the positive regulation of *AG* (this work). Taking into consideration the promiscuity of RNA-binding proteins, it is very plausible that components of the HUA-PEP activity might be participating in functionally distinct complexes. This is not unprecedented. For example Arabidopsis SR (serine/arginine rich) factors and the hnRNP AtGRP8 exhibit antagonistic and cooperative effects during circadian regulation [75]. Also, closely related MADS-box genes *AGAMOUS-LIKE 24* (*AGL24*) and *SHORT VEGETATIVE PHASE* (*SVP*) accelerate and delay flowering, respectively. Later, *AGL24* and *SVP* cooperate with *AP1* to downregulate *AG* during first stages of floral development [76–78]. Similarly, FUL-SVP replaces FLC-SVP heterodimers counteracting the repressive effect of the latter on flowering time [79]. Moreover, AG and AP3/PI participate in the same protein complexes to specify stamen anlagen. However, many genes promoting carpel development that are induced by *AG* are, on the contrary, repressed by *AP3*/*PI* [80]. Functional versatility of the *HUA-PEP* activity, in turn, might be very advantageous to provide regulatory flexibility to modulate the highly dynamic and complex networks governing reproductive development.

### Association of HUA-PEP proteins

PEP and FLK physically associate, as well as with HUA1 and HEN4, indicating that, probably, they all participate in common regulatory complexes. HUA2, however, might affect *AG* independently since no physical interaction between HUA2 and any other HUA-PEP component described here could be detected in a recent Y2H screen [81]. Formally, HUA2 molecular interactions might be mediated through HUA-PEP factors yet to be identified. We observed stronger phenotypes in *hua-pep* backgrounds when *HUA2* was mutated. These results might be explained with the existence of two complementary subactivities: one incorporating the HUA2 function and another one comprising the remaining identified HUA-PEP factors. Simultaneous disruption of both complexes might account for more profound phenotypic defects. Lethality in *hua2 pep* mutants substantiates this notion.

### PEP and FLK secure *AG* expression by mediating correct RNA processing

Our molecular analyses of *hua-pep* mutants are coincident with previous work showing accumulation of transcripts retaining intronic sequences at the expense of the functional *AG* mRNA [28]. A large intron where important regulatory motifs reside is a feature shared by *AG*, *FLC* and other MADS-box genes, that is conserved across species [82–87]. However, nascent transcripts are vulnerable to premature processing and large introns might increase the risk of cryptic signals recognizable by the splicing and/or polyadenylation machineries [88,89].

**Fig 7:**
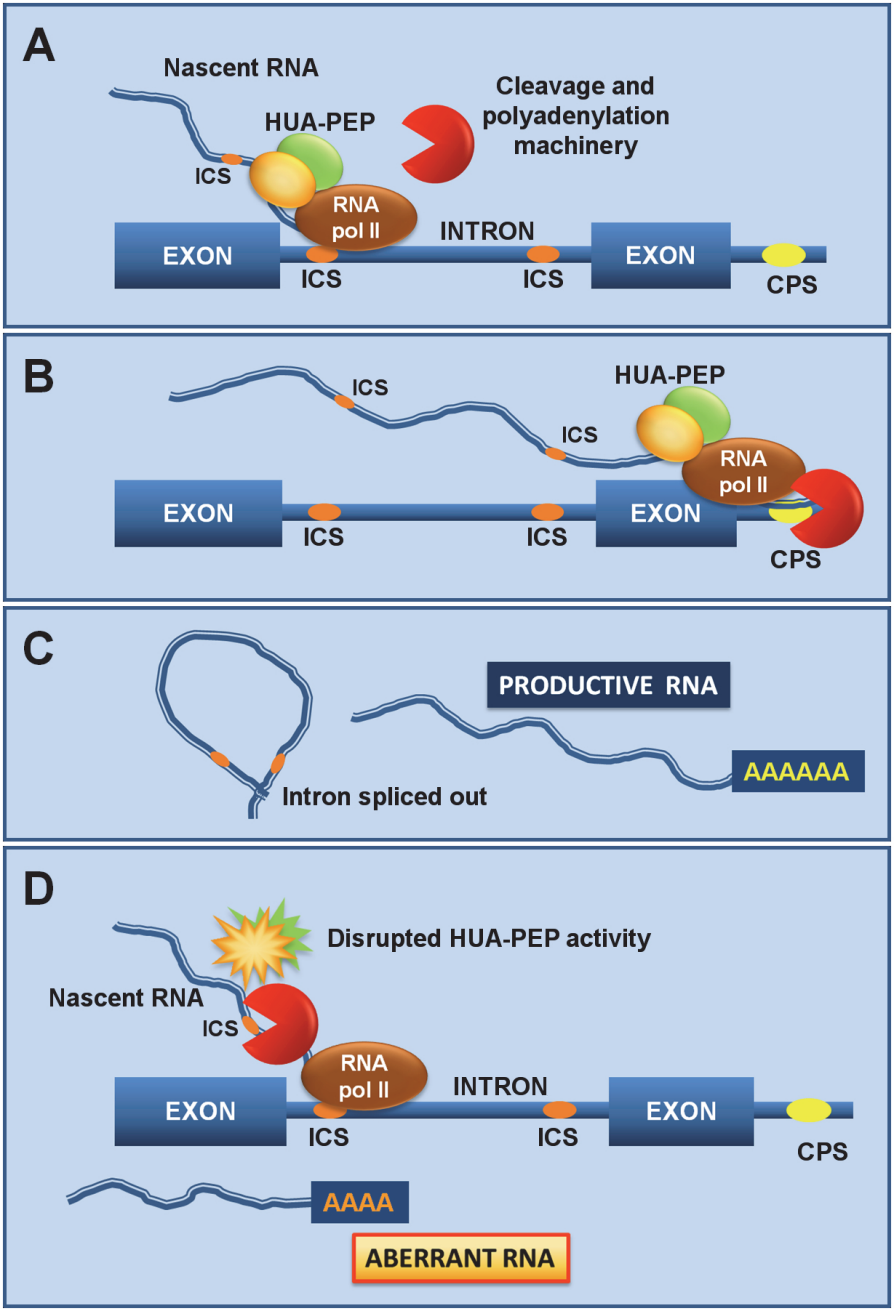
The HUA-PEP activity facilitates pre-mRNA processing of target genes. A) As the RNA polymerase (RNA pol II) activity progresses, the HUA-PEP hnRNP complex coats the nascent transcript, still chromatin-associated, thus sequestering intronic cryptic sites (ICS) from cleavage and polyadenylation. B) The elongation complex reaches the distal terminal cleavage and polyadenylation site (CPS), where correct termination occurs. C) Adequate intron excision and 3’ maturation take place. D) Conversely, an altered HUA-PEP activity does not prevent the RNA 3’ processing machinery to access cryptic motifs in the elongating transcript, producing thereby a prematurely terminated and ineffective RNA.

Transcript maturation mainly proceeds co-transcriptionally, increasing the fidelity of the process [24,90,91]. Altering *PEP* and *FLK* expression in the *hua1 hua2* background had a profound effect on the accumulation of *AG* intron-retaining transcripts. Remarkably, *FLC* intron-containing transcripts also increased in *pep* plants [46]. We propose that the HUA-PEP proteins assist transcription elongation by “hiding” cryptic signals in the nascent RNA (Fig. 7A-C). Otherwise, these sites could be accessible to the corresponding processing machinery, giving rise to non-functional or prematurely terminated transcripts (Fig. 7D). Our hypothesis is consistent with the recent characterization of mammal PCBPs as global regulators of alternative polyadenylation. Knock down of PCBPs actually favors usage of cryptic intronic sites [40]. Interestingly, hnRNP K suppresses usage of a premature polyadenylation site for *NEAT1*, a long non coding RNA (lncRNA) operating in nuclear paraspeckles (ribonucleoprotein bodies) formation, thus increasing the ratio of the long effective transcript [92].

By sequestering intronic polyadenylation motifs, PEP (and the remaining HUA-PEP factors) may also facilitate correct splicing, as documented for other PCBPs [36,41]. The U1 snRNP (U1), in addition to its splicing role, protects pre-mRNAs from premature termination at intronic polyadenylation sites [88,89], raising the attractive possibility of a connection with the *HUA-PEP* gene activity. The carboxyl-terminal (CTD) domain of eukaryotic RNA polymerase II coordinates transcription and transcript maturation [93]. The Arabidopsis KH protein SHINY1 (SHI1) interacts with a phosphatase that dephosphorylates particular residues in CTD, downregulating transcription of abiotic stress-related genes by preventing 5’ capping [94,95]. Uncovering new functional and molecular relationships among distinct *HUA-PEP* components will certainly provide a better understanding of the developmental programs regulated by this activity (floral timing; flower patterning) and the importance, at the regulatory level, of multifunctional plant PCBP-type hnRNPs.

## Materials and Methods

### Plant material

This work was carried out with the *Arabidopsis thaliana* Columbia (Col-0) accession as the wild type. Strains previously obtained in other accessions were backcrossed at least five times into Col-0 before any further experiment. Plant materials used in this study were *pep-4* [44], *flk-2* [43], *hua2-4* [73]; *hua2-7* [74], *35S*::*PEP* [46], and *ful-1* [51]. *gAG*::*AG-GFP* and *gAP1*::*AP1-GFP* [62] were provided by Gerco Angenent and Richard Immink (Wageningen University, The Netherlands). *hen4-2* [28], *hua1-1* and *hua2-1* [30] were provided by Xuemei Chen (UC Riverside, USA). *KNU*::*GUS* [16] was provided by Anna M. Koltunow (CSIRO, Adelaide, Australia). Information about all primers used in this work and molecular genotyping can be found in S2 Table. Plants were grown in MS plates or soil as previously described [44].

### Microscopy and histology

Phloroglucinol lignin staining [96,97] and GUS assays were performed essentially as described [44,96,97]. All GUS analyses, except in the case of *ful-1/+*, were performed in homozygous lines. Whole-mount pictures were taken under a Nikon SMZ1500 stereomicroscope. Histological sections (8 μm) were photographed under bright-field or dark-field illumination using a Nikon E800 microscope. In both cases Nikon Digital Camera DXM1200F was used operated by the ACT-1 2.70 program. Scanning electron microscopy (SEM) was according to [44]. For confocal laser scanning microscopy, all analyses were performed in homozygous lines. Samples were pre-treated with methanol/acetone (1:1 v/v) solution for 30 minutes at -20°C, and subsequently rinsed in PBS buffer (1.94 mM K_2_PO4; 8.06 mM Na_2_PO4; 2.7 mM KCl, 0.137 mM NaCl, pH 7.4) to be observed under a Leica TCS SPE confocal microscope. Pictures were taken with the LAS AF program.

### Quantitative RT-PCR and RACE

For quantitative RT-PCR (qPCR), 5 μg of total RNA was extracted from young flower buds until stage 9, treated with DNase I, and used for cDNA synthesis with an oligo(dT) primer and RevertAid Premium Reverse Transcriptase (Thermo Scientific) following the manufacturer’s instructions. Subsequently, for each qPCR reaction, 0.5 μl of the cDNA was used as template. Relative changes in gene expression levels were determined using the LyghtCycler 1.5 system with the LightCycler FastStart DNA amplification kit according to the manufacturer (Roche Diagnostics). RNA levels were normalized to constitutively expressed genes *OTC* (*ORNITINE TRANSCARBAMILASE*) [98] and *ACT2* [99], and the corresponding wild-type levels, as previously reported [46,100]. Each experiment was undertaken using three biological replicates with three technical replicates each. The standard deviation was calculated in Microsoft Excel. Statistical significance was estimated by the Student’s *t*-test according to [101] (*P < 0.05, **P < 0.01).

For 3’ rapid amplification of cDNA ends (3’ RACE), 5 μg of young flower bud total RNA was reverse transcribed using Maxima Reverse Transcriptase and the adaptor oligo d(T)-anchor (kit 5’/3’ RACE, Roche Diagnostics) as a primer. Then, *AG* cDNAs were amplified with High Fidelity PCR Enzyme Mix (Thermo Scientific) using forward primers situated in the exon 2 (S2 Table) and the PCR anchor (Roche Diagnostics) as a reverse primer hybridizing with the adaptor sequence, thus ensuring that only polyA-containing sequences were amplified. Amplified products were cloned into pSC-A plasmids and sequenced with M13F and M13R primers. Sequences were analyzed using CLUSTAL-W aligning [102].

### Protein interactions

For bimolecular fluorescence complementation (BiFC), coding sequences of all genes under study were amplified from their respective cDNAs using Phusion Taq-polymerase (NEB). The corresponding primer sequences (S2 Table) were designed for cloning the resulting PCR amplicons via Gibson DNA assembly method [103], and cloned into both the pBJ36-SPYNE and pBJ36-SPYCE plasmids, containing N-terminal (nt) and C-terminal (ct) halves of the yellow fluorescent protein (YFP), respectively (YFPnt and YFPct) [104]. The *35S*::*SPYNE* and *35S*::*SPYCE* cassettes were then cloned via *Not*I into the binary vectors pGreen0229 and pGreen0179 [105], respectively. Transformed AGL-0 *Agrobacterium tumefaciens* cells were used to infect *Nicotiana benthamiana* leaves. YFP reconstituted fluorescence was visualized 72 h after inoculation under a Nikon Eclipse TE2000-U epifluorescence microscope. The reciprocal BiFC assays were also performed obtaining the same results as shown in Fig. 6 and S13 Fig. As negative controls, *Nicotiana* leaves were co-infiltrated with the corresponding recombinant YFPct construct and the empty YFPnt version, yielding no signal in any case.

For yeast two-hybrid assays, the cDNAs for *PEP*, *FLK*, *HEN4* and *HUA1* genes were amplified with the proof-reading Phusion Taq-polymerase (New England Biolabs, Inc.) using the corresponding primers (S2 Table). The resulting products were cloned into the pB42AD (+Trp) and pGilda (+His) vectors via Gibson DNA assembly procedure [103]. The integrity of constructs was checked by sequencing. The yeast strain EGY48 (-Ura) was cotransformed with the corresponding combinations of *pGilda* and *pB42AD* constructs. Empty vectors were used as negative controls. Positive colonies were selected on solid media (-Ura, -His, -Trp +glucose). Induction for testing protein-protein association was assayed growing the resulting yeast strains on plates or liquid in the presence of galactose and raffinose (DB Falcon). X-gal was used for colorimetric assays on plates, and ONPG (2-Nitrophenyl β-D-galactopyranoside, SIGMA) for β-galactosidase liquid experiments. The Clontech protocol book was followed for all these procedures.

## Supporting Information

S1 Fig Flower phenotypes of single mutants and mutant combinations between *pep* and *hen4*, *hua1* and *hua2*. A-C) Flowers and young pollinated pistils of *hen4*, *hua1* and *hua2–7*, respectively. Front sepals and petals were manually removed to show wild-type looking stamens. D) Siliques/gynoecia from wild-type (WT) and different *hua-pep* mutants. Scale bars: 1 mm.
(TIFF)

S2 Fig Additional flower phenotypes of mutant combinations between *pep* and *hen4*, *hua1* and *hua2*. A) Post-anthesis wild-type flower after removing some outer organs. B-D) SEMs of the adaxial side of a *hen4 pep* anther partially transformed into a petal-like organ. The transformed organ retains staminoid features, and even pollen production (B). The apical portion shows petaloid histology (C), whereas normal anther cells occur at the base (D). E, F) Top portion of apically open *hua1 pep* (E) and *hua1 hen4 pep/+* (F) pistils. Observe little stigmatic development, absent style, and white pointed tip (arrow). G) SEM of the apical portion of a *hua1 hua2 pep/+* gynoecium. H-K) SEMs of wild-type valve epidermal layer (H), close-up view of *hua1 pep* valve territory showing irregular striated cells (I) and wild-type sepal (J, K). L) *hua1 pep* pistil with supernumerary valves (arrows), open at the top and containing residual style and stigma tissues. M) A *hua1 pep* gynoecium. A fourth whorl organ was manually removed to show developing floral organs inside (white arrow) together with normal ovules (yellow arrow). N) SEM of a *hen4 hua1 pep/+* gynoecium displaying an extra valve (white arrow). O) A *hua1 hua2 pep/+* gynoecium in which a valve-like organ was manually removed to show additional flower organs inside. P-R) Flower phenotypes of *hua1 hen4 hua2/+ pep/+* plants. Scale bars: 1 mm (A, F, L-R), 500 μm (E), 100 μm (B, G, J), 10 μm (H, I, K) and 2 μm (C,D).
(TIFF)

S3 Fig *hua2-4* is a leaky allele. Expression levels of *HUA2* mRNA from 16-day-old wild-type (WT, Col-0) and *hua2-4* mutant individuals monitored by quantitative RT-PCR (qRT-PCR). Error bars, standard deviation (SD).
(TIFF)

S4 Fig Fruit phenotypes resulting of combining *hua-pep* mutants with the *ful* background. A) SEM images of fruits/gynoecia from *ful* and *hua-pep* mutant combinations. In *ful hua2* and *ful pep* plants the style was shortened and stripes of longer cells occurred on the valve, likely explaining a modest increase in size with respect to *ful* fruits. This trend was enhanced in the *ful hua1 pep* triple mutant. These plants produced longer fruits with thinner repla (r) and further reduction of the style territory. In *ful hua1 hua2* these latter traits were even further pronounced but gynoecia were smaller due to associated fertility problems. In *ful hua1 hua2 pep/+*, where long gynophores are common, gynoecia appear bulged as a consequence of additional floral organs growing inside. B, C) Details of valve territory of *ful hua2* (B) and *ful hua1 hua2* (C) flowers. D, E) Whole-mount *FUL* GUS-staining (*ful-1*) in *ful/+* wild-type looking pistil (D) and *ful hua1 hua2 pep*/+ gynoecium (E). F-I) *FUL* GUS staining in cross-sections of wild-type sepal (F) and fully developed fruits of *ful* (G), *ful hua1 hua2* (H), and *ful hua1 hua2 pep/+* (I) mutants. In *ful*, characteristic valve staining (G) can be appreciated. In (H) and (I) GUS signal is faint, essentially coinciding with the vasculature. Big cells (bc) appear on the outer surface (H), like in sepals (F). Inner additional floral organs (fo) can be seen in *ful hua1 hua2 pep/+* (I). J-N) Whole-mount lignin-specific staining with phloroglucinol. Mature wild-type (J) and *ful* (K) siliques. In wild-type red phloroglucinol staining is limited to dehiscence zones whereas in *ful* staining is detected in valves due to the ectopically lignification of this territory. In *ful hua1 hua2 pep/+* (L, M), lignification is largely coincident with the vascular system, closely resembling that of a wild-type sepal (N). Dissection of the fourth whorl reveals the presence of additional floral organs inside (M). Observe staining domains connected to basal structures as the long gynophore and the floral stalk (M, red arrows). Scale bars: 1 mm (A, D, E, J-M), 100 μm (B, C, F-I) and 500 μm (N).
(TIF)

S5 Fig Expression of *AP1* and *PI* genes in *hua-pep* mutant backgrounds. Relative mRNA expression of *AP1* (A) and *PI* (B) in the wild type (WT) and diverse mutant backgrounds, monitored by qPCR. Error bars, SD. Asterisks indicate statistically significant differences from *hua1 hua2* plants (*P <0.05, **P < 0.01).
(TIFF)

S6 Fig The mRNA expression of *SPL* and *KNU* is regulated by the *HUA-PEP* activity. A, B) mRNA expression levels of *SPL* (A) and *KNU* (B), respectively, in the wild type (WT) and diverse *hua-pep* mutant backgrounds, monitored by qPCR. Error bars, SD. C-H) *KNU*::*GUS* [16] expression pattern in wild-type and *hua1 pep* mutant backgrounds. Longitudinal sections (C, D, F, G) and whole-mount (E, H) staining. C) Stage 6 wild-type flower showing GUS signal in a central spot between growing carpel primordia, as previously reported [16]. F) No detectable GUS signal was seen in stage 6 *hua1 pep* flowers. D) In wild-type stage 8 flowers strong staining also appears in stamens (white arrows). G) Stage 8 *hua1 pep* flower. GUS signal is absent at the base of the fourth whorl and substantially less intense in stamens (yellow arrows). E) Apex of a wild-type pistil during stage 12. *GUS* expression is detectable in ovules and internal sections of the style. H) Stage 12 *hua1 pep* pistil showing a significant reduction in reporter expression (yellow line). Scale bars: 50 μm.
(TIFF)

S7 Fig Increasing *AG* gene-dosage rescues the wild-type floral phenotype in the *hua1 pep* mutant. A) *hua1 pep* mutant flower in which sepals and petals have been manually removed to show petaloid conversions of stamens (red arrow). B) A heavily distorted *hua1 pep* gynoecium. C, D) Flower and silique, respectively, of a triple homozygous *hua1 pep gAG*:*GFP* plant. In (C) some outer organs were manually removed to visualize wild-type looking organs inside. Scale bars: 1mm.
(TIFF)

S8 Fig Detection of the AP1-GFP fusion protein in wild-type plants by confocal microscopy. A) Inflorescence showing signal in sepals. B) Magnification of one of the flowers from (A). C) AP1-GFP signal in stage 12 pistil. Autofluorescence was arbitrarily displayed in blue or red. Scale bars: 75 μm (A,C) and 25 μm (B).
(TIFF)

S9 Fig Reproductive phenotypes of *PEP* overexpression in the *hua1 hua2* and *hen4 hua1* backgrounds. A-E) SEM images. Adaxial surface of wild-type anther (A) and petal (B), and *hua1 hua2 35S*::*PEP* third whorl organ (C). In *hua1 hua2 35S*::*PEP* style and stigma were dramatically reduced and apical closure usually failed (D), as well as flower determinacy (E). F-J) Flower phenotypes of *hua1 hen4 35S*::*PEP* recapitulating the mutant traits appearing in the previous genotype. K, L) Relative expression levels of *SPL* (K) and *KNU* (L) mRNA in the wild type (WT) and diverse mutant backgrounds, monitored by qPCR. M) Scale bars: 10 μm (A-C), 100 μm (D, J), 1 mm (E-H) and 500 μm (I). Error bars, SD.
(TIFF)

S10 Fig The floral phenotypes of *hua1 hua2 35S*::*PEP* plants are not the result of *PEP* silencing. A) Double homozygous *hen4 35S*::*PEP* flower. B) Double homozygous *hua1 35S*::*PEP* flower. C) Double homozygous *hua2 35S*::*PEP* flower. D) *PEP* mRNA levels were monitored by qPCR in the wild type and *hua1 hua2-4* plants hemizygous (*hua1 hua2-4 35S*::*PEP/+*) or homozygous (*hua1 hua2-4 35S*::*PEP*) for the *35S*::*PEP* construct, respectively. Transgenic plants produced much higher levels of *PEP* transcript than the wild type. We used the *hua2-4* allele [73] (SALK_032281) containing caulimoviral 35S promoter sequences, potentially able to trigger silencing of overexpressor lines driven by the same promoter element [Daxinger et al., 2008 in S2 Table]. E-G) The phenotype of *hua1 hua2-4* gynoecia (E) was not modified in *hua1 hua2-4 35SPEP/+* plants (F), but appeared dramatically enhanced in triple homozygous *hua1 hua2-4 35S*::*PEP* plants (G). H-J) Similar results were obtained with the *hua2-7* allele [74] (SAIL_314_A08) lacking 35S sequences in its T-DNA. H) *hua1 hua2-7* gynoecium. I) *hua1 hua2-7 hua1 hua2-7 35S*::*PEP/+* gynoecium. J) *hua1 hua2-7 35S*::*PEP* gynoecium. In panels (A-C) some outer organs were manually removed to visualize inner organs. Scale bars: 1 mm. Error bars, SD.
(TIFF)

S11 Fig Sequence scheme of prematurely processed *AG* transcripts identified by RACE. DNA sequence corresponding to exon 2 appears as white upper-case letters boxed in black. Intron 2 sequence is shown as lower-case black letters. Cleavage site is indicated (A in red). Putative cis-elements associated to cleavage and polyadenylation are depicted boxed in blue (FUE), green (NUE) and yellow (CE), respectively [63]. A) Some prematurely processed transcripts identified in the *hua1 hua2 pep/+* mutant background. B) An aberrantly processed transcript identified in the *hua1 hua2 35S*::*PEP* mutant background. The most abundant version of FUE in plants (UUUGUU boxed in blue), and a canonical AAUAAA sequence element for NUE (green) are indicated [63]. C) Prematurely terminated transcripts identified in the *flk hua1 hua2* mutant background.
(TIFF)

S12 Fig Interactions of *flk* with other *hua-pep* mutants. Floral phenotypes and RNA expression data. A) Wild-type looking *flk hen4* double mutant flower. Some outer organs were manually removed to show normal looking stamens. B) Young developing *flk hen4* fruit. C) Wild-type looking *flk hua1* double mutant flower. Some outer organs were manually removed to show normal looking stamens. D-F) SEM images of *flk pep hua1/+* flower organs. D) Corrugated pistil. E) A flower after removing most organs around the pistil, except three third whorl organs with obvious petaloid traits. F) Close-up view of the adaxial surface of a petaloid area in a third whorl organ in (E) showing conically shaped cells. G, H) SEM images of *flk hua1 hua2* flower organs. G) Petaloid conically shaped cells on the adaxial surface of a third whorl organ. H) A flower in which a fourth whorl organ was manually removed to uncover additional flowers inside. I) Relative mRNA expression of *SPL* and *KNU* in the wild type (WT) and diverse mutant backgrounds, monitored by qPCR. Error bars, SD. Scale bars: 1 mm (A, B, C, H), 200 μm (E, F), 10 μm (D, G).
(TIFF)

S13 Fig Additional transient BiFC experiments. Visualization of YFP reconstitution (yellow fluorescence) in *Nicotiana benthamiana* leaf cells agroinfiltrated with plasmids encoding fusion proteins. The first 5 interactions show the reciprocal assays of those depicted in Fig. 6 (the first protein fused to the YFPct, and the second protein to the YFPnt). The interaction between HEN4 and HUA1 [28] was confirmed in both directions and used as a positive control. The ability of PEP, FLK and HUA1 to homodimerize was also verified. HEN4 was unable to homodimerize, thus providing a negative control. As further negative controls, *Nicotiana* leaves were co-infiltrated with the corresponding recombinant YFPct construct and the empty YFPnt version. The reciprocal assays were also performed. No signal was detected in any case. In merged visible+YFP fluorescence pictures, blue background has been used to increase the contrast.
(TIFF)

S14 Fig Yeast two hybrid assays to test protein-protein interactions between PEP, FLK, HEN4 and HUA1. Protein interactions for PEP (A), FLK (B), and HUA1 (C). Induced yeast X-Gal plate assays are shown on the top of each panel. Y2H liquid assays using the reagent ONPG are shown below. Error bars indicate standard deviation (SD).
(TIFF)

S1 Table Loss and overexpression of *PEP* enhance the mutant phenotypes of *hua1 hua2* flowers. For each genotype approximately 75 flowers from 5 different plants were examined. Flowers were collected from top, bottom and intermediate zones of each plant. Petaloid transformations of stamens were arbitrarily classified as mild (totally or partially transformed lateral stamens), intermediate (transformed lateral stamens and partially converted medial stamens), and strong (all indistinguishable from second whorl petals). In *hua1 hua2*, pistils with four valves were extremely scarce whereas in the other two genotypes were easily found.
(DOCX)

S2 Table Oligonucleotides, genotyping and additional references. (DOCX)
