# Supplementary figures and images for: K-homology Nuclear Ribonucleoproteins Regulate Floral Organ Identity and Determinacy in Arabidopsis
Source: PLoS Genet. 2015 Feb 6;11(2):e1004983. doi: 10.1371/journal.pgen.1004983 (PMC4450054; doi:10.1371/journal.pgen.1004983)

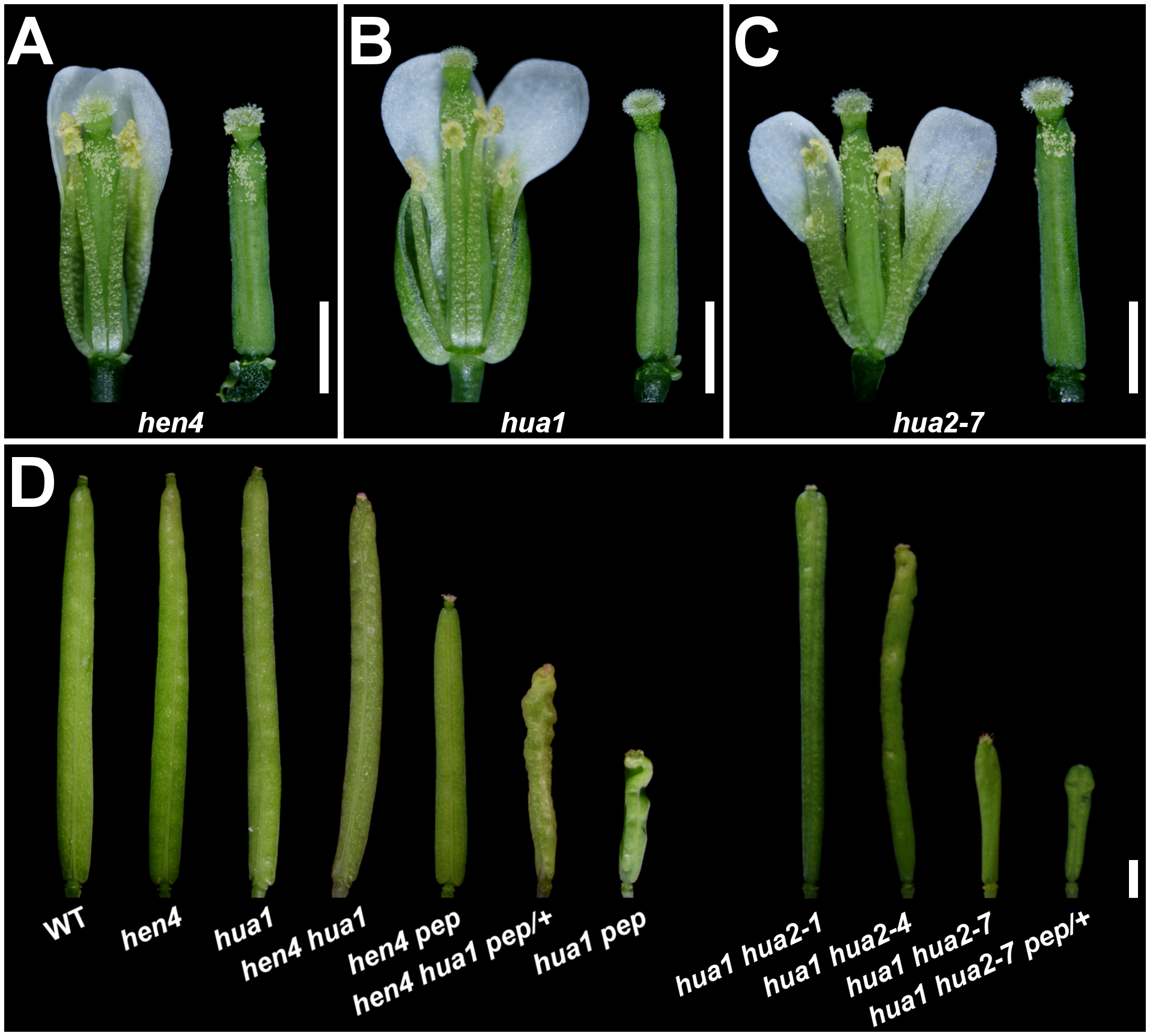

Supplement: S1 Fig — A-C) Flowers and young pollinated pistils of hen4, hua1 and hua2–7, respectively. Front sepals and petals were manually removed to show wild-type looking stamens. D) Siliques/gynoecia from wild-type (WT) and different hua-pep mutants. Scale bars: 1 mm. (TIFF) [file pgen.1004983.s001.tiff]

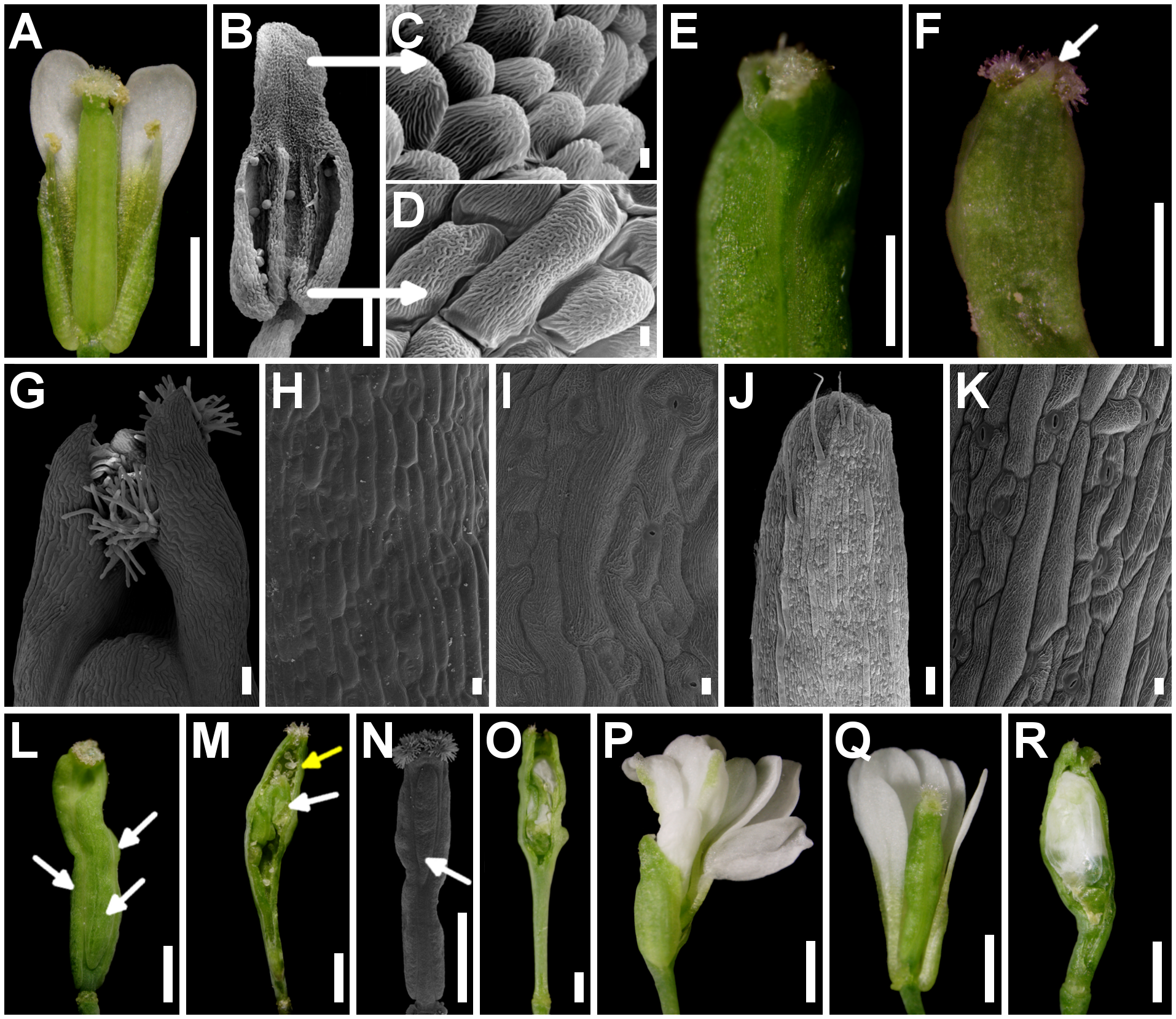

Supplement: S2 Fig — A) Post-anthesis wild-type flower after removing some outer organs. B-D) SEMs of the adaxial side of a hen4 pep anther partially transformed into a petal-like organ. The transformed organ retains staminoid features, and even pollen production (B). The apical portion shows petaloid histology (C), whereas normal anther cells occur at the base (D). E, F) Top portion of apically open hua1 pep (E) and hua1 hen4 pep/+ (F) pistils. Observe little stigmatic development, absent style, and white pointed tip (arrow). G) SEM of the apical portion of a hua1 hua2 pep/+ gynoecium. H-K) SEMs of wild-type valve epidermal layer (H), close-up view of hua1 pep valve territory showing irregular striated cells (I) and wild-type sepal (J, K). L) hua1 pep pistil with supernumerary valves (arrows), open at the top and containing residual style and stigma tissues. M) A hua1 pep gynoecium. A fourth whorl organ was manually removed to show developing floral organs inside (white arrow) together with normal ovules (yellow arrow). N) SEM of a hen4 hua1 pep/+ gynoecium displaying an extra valve (white arrow). O) A hua1 hua2 pep/+ gynoecium in which a valve-like organ was manually removed to show additional flower organs inside. P-R) Flower phenotypes of hua1 hen4 hua2/+ pep/+ plants. Scale bars: 1 mm (A, F, L-R), 500 μm (E), 100 μm (B, G, J), 10 μm (H, I, K) and 2 μm (C,D). (TIFF) [file pgen.1004983.s002.tiff]

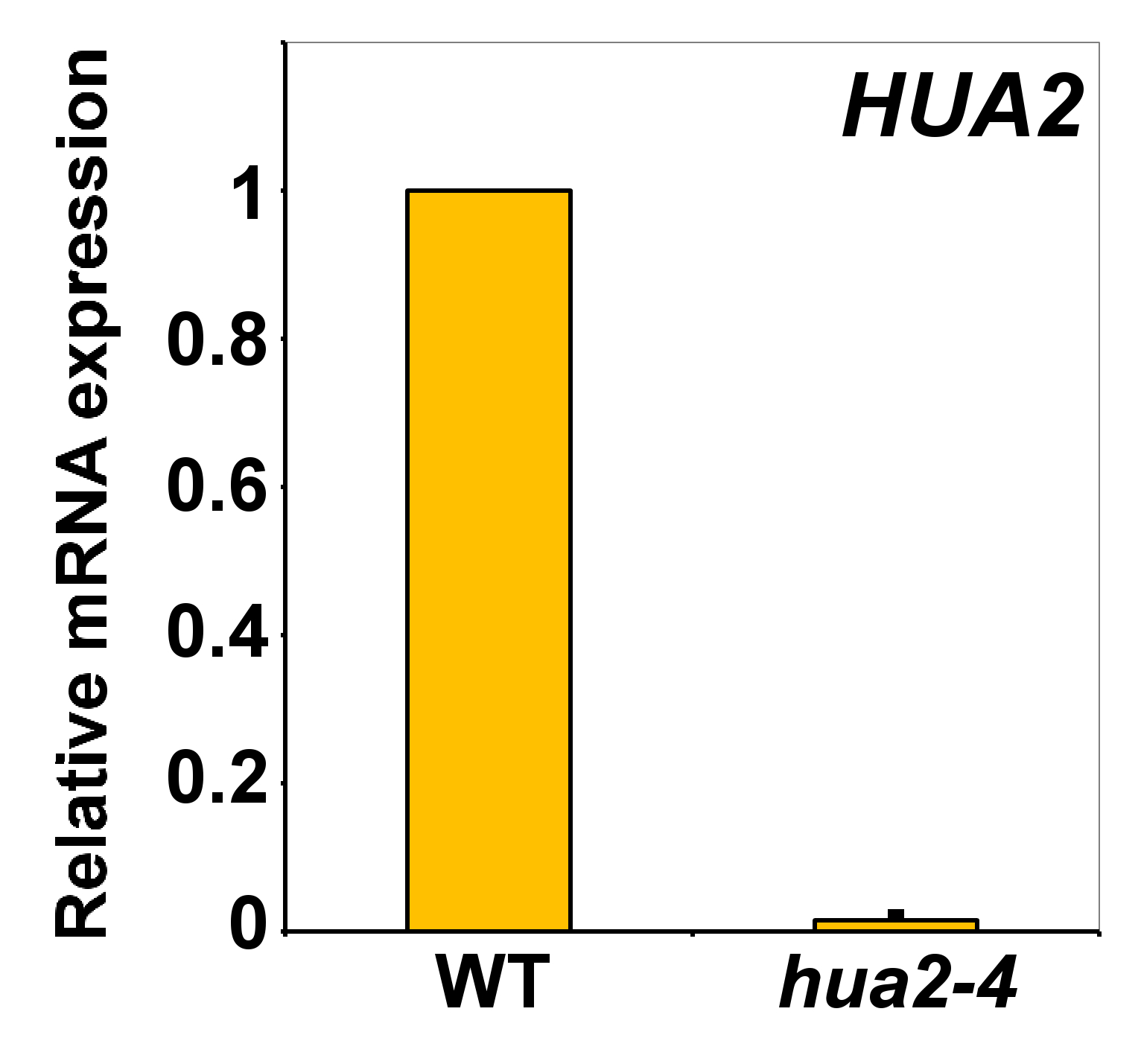

Supplement: S3 Fig — Expression levels of HUA2 mRNA from 16-day-old wild-type (WT, Col-0) and hua2-4 mutant individuals monitored by quantitative RT-PCR (qRT-PCR). Error bars, standard deviation (SD). (TIFF) [file pgen.1004983.s003.tiff]

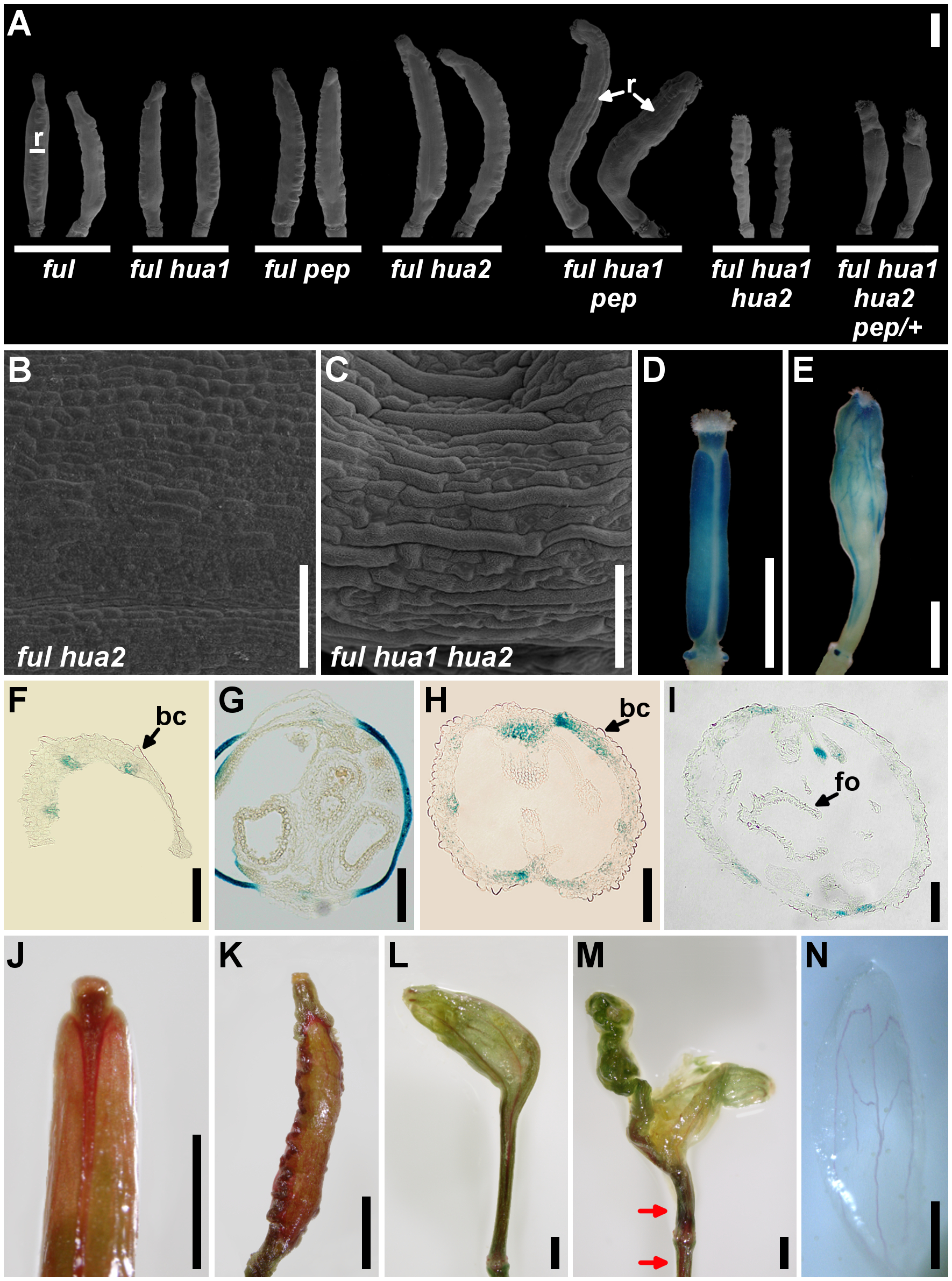

Supplement: S4 Fig — A) SEM images of fruits/gynoecia from ful and hua-pep mutant combinations. In ful hua2 and ful pep plants the style was shortened and stripes of longer cells occurred on the valve, likely explaining a modest increase in size with respect to ful fruits. This trend was enhanced in the ful hua1 pep triple mutant. These plants produced longer fruits with thinner repla (r) and further reduction of the style territory. In ful hua1 hua2 these latter traits were even further pronounced but gynoecia were smaller due to associated fertility problems. In ful hua1 hua2 pep/+, where long gynophores are common, gynoecia appear bulged as a consequence of additional floral organs growing inside. B, C) Details of valve territory of ful hua2 (B) and ful hua1 hua2 (C) flowers. D, E) Whole-mount FUL GUS-staining (ful-1) in ful/+ wild-type looking pistil (D) and ful hua1 hua2 pep/+ gynoecium (E). F-I) FUL GUS staining in cross-sections of wild-type sepal (F) and fully developed fruits of ful (G), ful hua1 hua2 (H), and ful hua1 hua2 pep/+ (I) mutants. In ful, characteristic valve staining (G) can be appreciated. In (H) and (I) GUS signal is faint, essentially coinciding with the vasculature. Big cells (bc) appear on the outer surface (H), like in sepals (F). Inner additional floral organs (fo) can be seen in ful hua1 hua2 pep/+ (I). J-N) Whole-mount lignin-specific staining with phloroglucinol. Mature wild-type (J) and ful (K) siliques. In wild-type red phloroglucinol staining is limited to dehiscence zones whereas in ful staining is detected in valves due to the ectopically lignification of this territory. In ful hua1 hua2 pep/+ (L, M), lignification is largely coincident with the vascular system, closely resembling that of a wild-type sepal (N). Dissection of the fourth whorl reveals the presence of additional floral organs inside (M). Observe staining domains connected to basal structures as the long gynophore and the floral stalk (M, red arrows). Scale bars: 1 mm (A, D, E, J-M), 100 [file pgen.1004983.s004.tif]

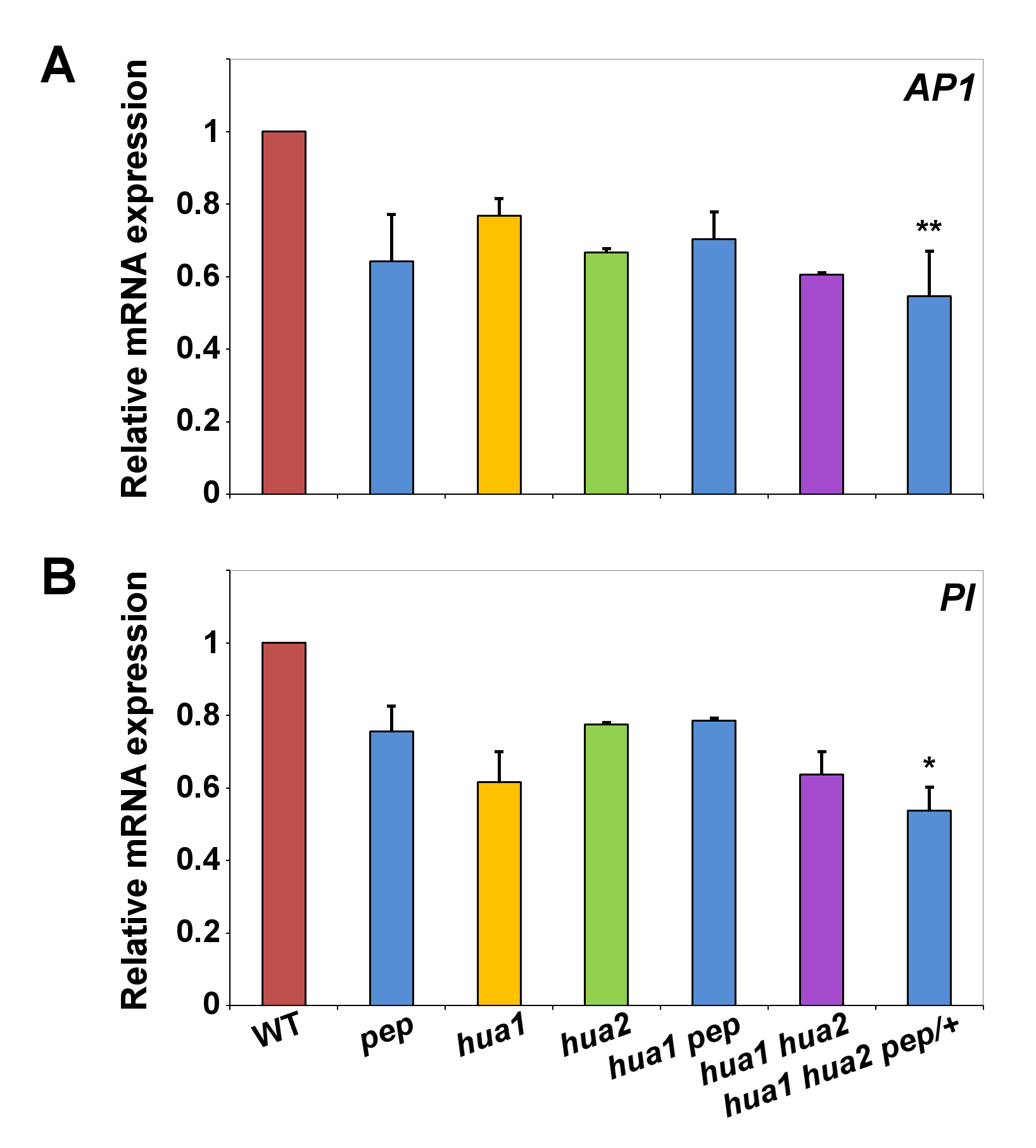

Supplement: S5 Fig — Relative mRNA expression of AP1 (A) and PI (B) in the wild type (WT) and diverse mutant backgrounds, monitored by qPCR. Error bars, SD. Asterisks indicate statistically significant differences from hua1 hua2 plants (*P <0.05, **P < 0.01). (TIFF) [file pgen.1004983.s005.tiff]

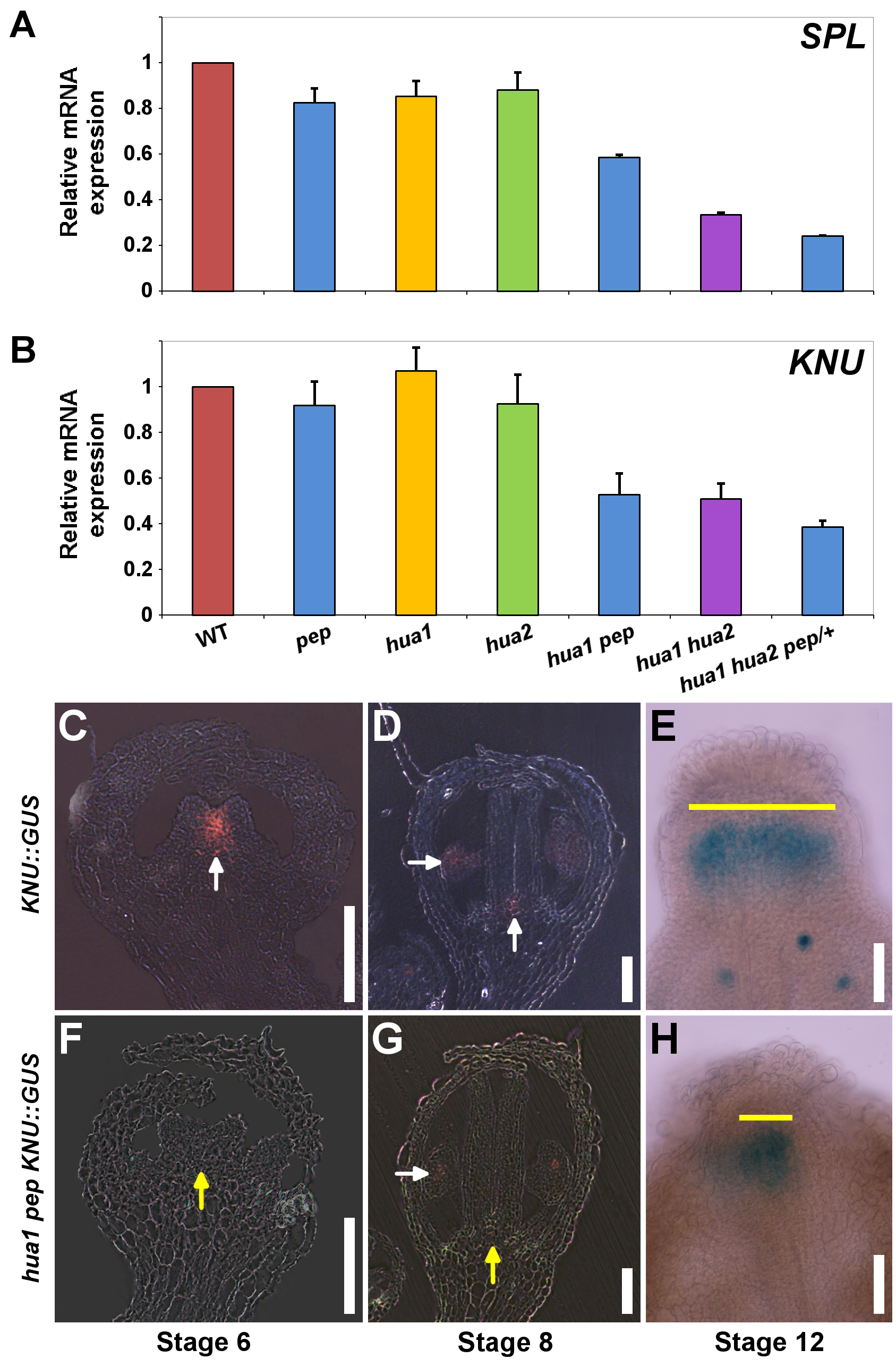

Supplement: S6 Fig — A, B) mRNA expression levels of SPL (A) and KNU (B), respectively, in the wild type (WT) and diverse hua-pep mutant backgrounds, monitored by qPCR. Error bars, SD. C-H) KNU::GUS [16] expression pattern in wild-type and hua1 pep mutant backgrounds. Longitudinal sections (C, D, F, G) and whole-mount (E, H) staining. C) Stage 6 wild-type flower showing GUS signal in a central spot between growing carpel primordia, as previously reported [16]. F) No detectable GUS signal was seen in stage 6 hua1 pep flowers. D) In wild-type stage 8 flowers strong staining also appears in stamens (white arrows). G) Stage 8 hua1 pep flower. GUS signal is absent at the base of the fourth whorl and substantially less intense in stamens (yellow arrows). E) Apex of a wild-type pistil during stage 12. GUS expression is detectable in ovules and internal sections of the style. H) Stage 12 hua1 pep pistil showing a significant reduction in reporter expression (yellow line). Scale bars: 50 μm. (TIFF) [file pgen.1004983.s006.tiff]

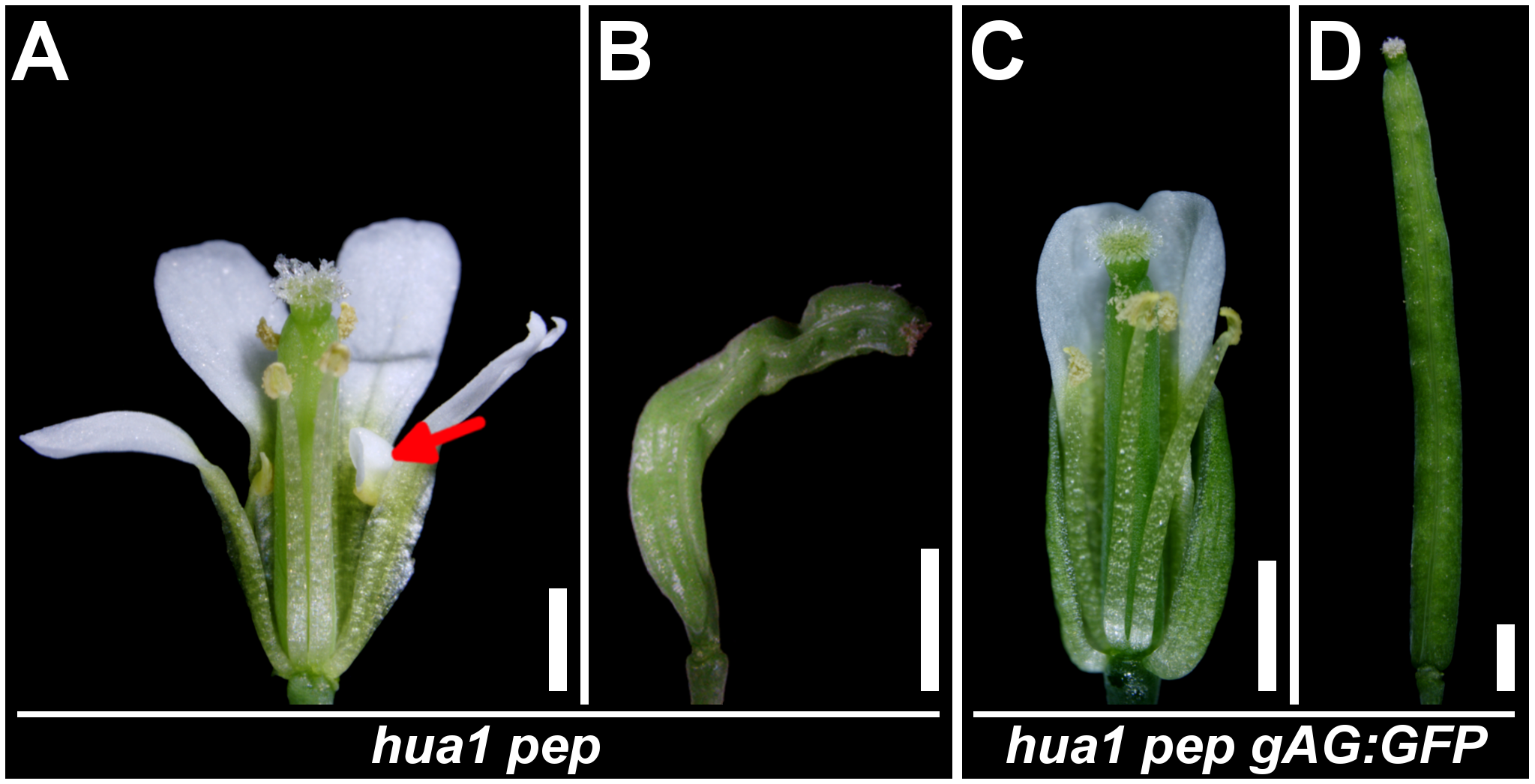

Supplement: S7 Fig — A) hua1 pep mutant flower in which sepals and petals have been manually removed to show petaloid conversions of stamens (red arrow). B) A heavily distorted hua1 pep gynoecium. C, D) Flower and silique, respectively, of a triple homozygous hua1 pep gAG:GFP plant. In (C) some outer organs were manually removed to visualize wild-type looking organs inside. Scale bars: 1mm. (TIFF) [file pgen.1004983.s007.tiff]

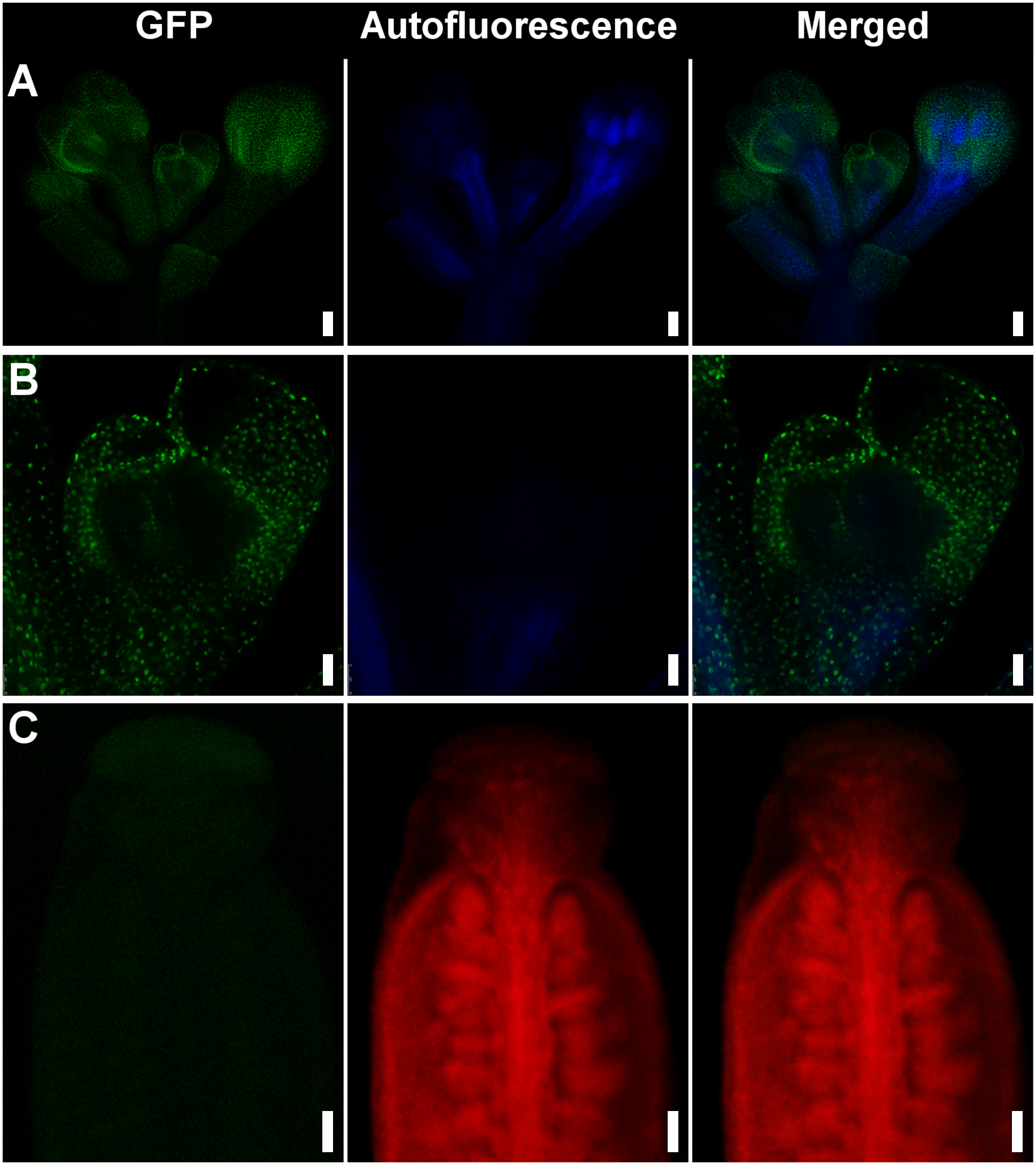

Supplement: S8 Fig — A) Inflorescence showing signal in sepals. B) Magnification of one of the flowers from (A). C) AP1-GFP signal in stage 12 pistil. Autofluorescence was arbitrarily displayed in blue or red. Scale bars: 75 μm (A,C) and 25 μm (B). (TIFF) [file pgen.1004983.s008.tiff]

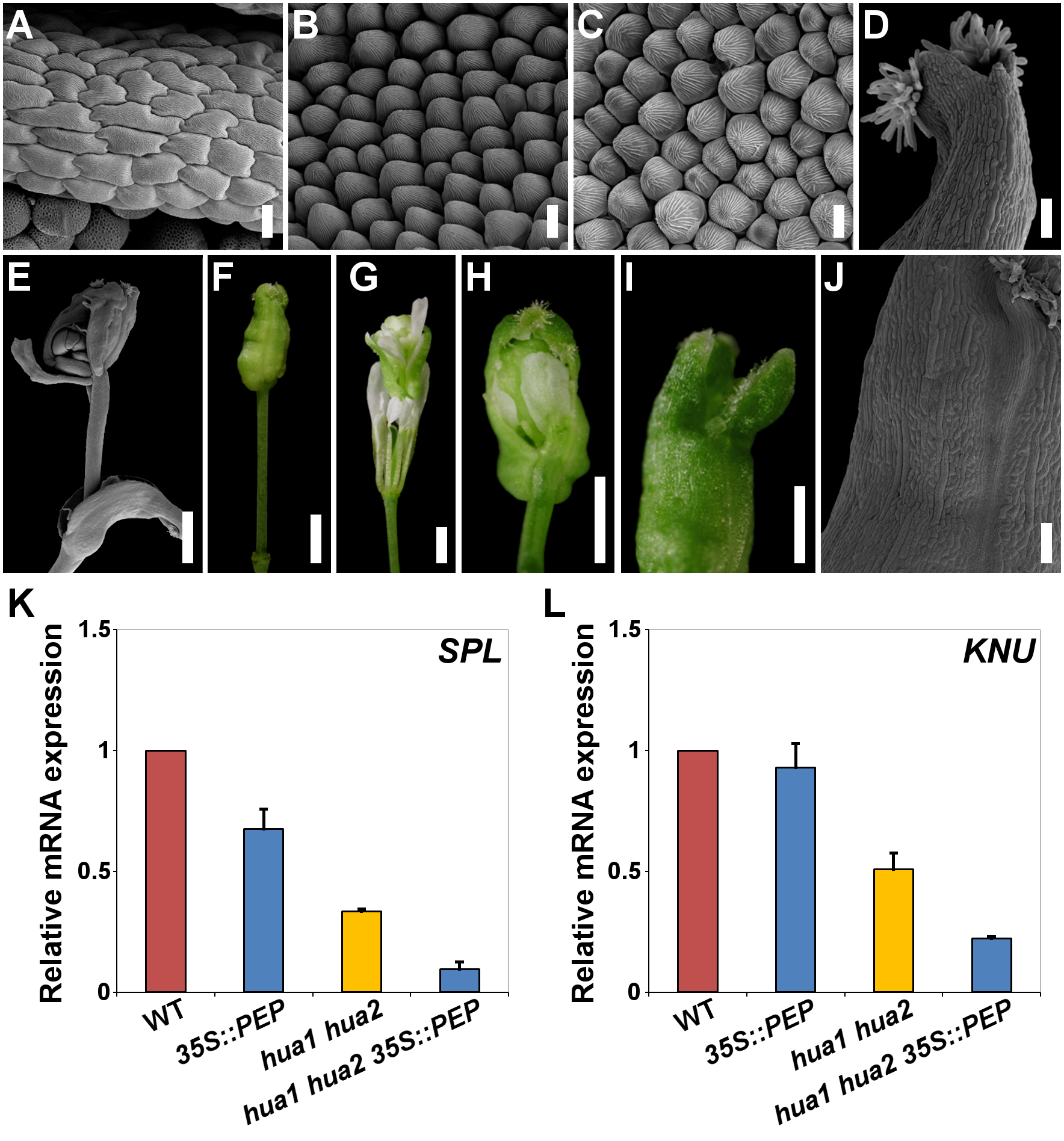

Supplement: S9 Fig — A-E) SEM images. Adaxial surface of wild-type anther (A) and petal (B), and hua1 hua2 35S::PEP third whorl organ (C). In hua1 hua2 35S::PEP style and stigma were dramatically reduced and apical closure usually failed (D), as well as flower determinacy (E). F-J) Flower phenotypes of hua1 hen4 35S::PEP recapitulating the mutant traits appearing in the previous genotype. K, L) Relative expression levels of SPL (K) and KNU (L) mRNA in the wild type (WT) and diverse mutant backgrounds, monitored by qPCR. M) Scale bars: 10 μm (A-C), 100 μm (D, J), 1 mm (E-H) and 500 μm (I). Error bars, SD. (TIFF) [file pgen.1004983.s009.tiff]

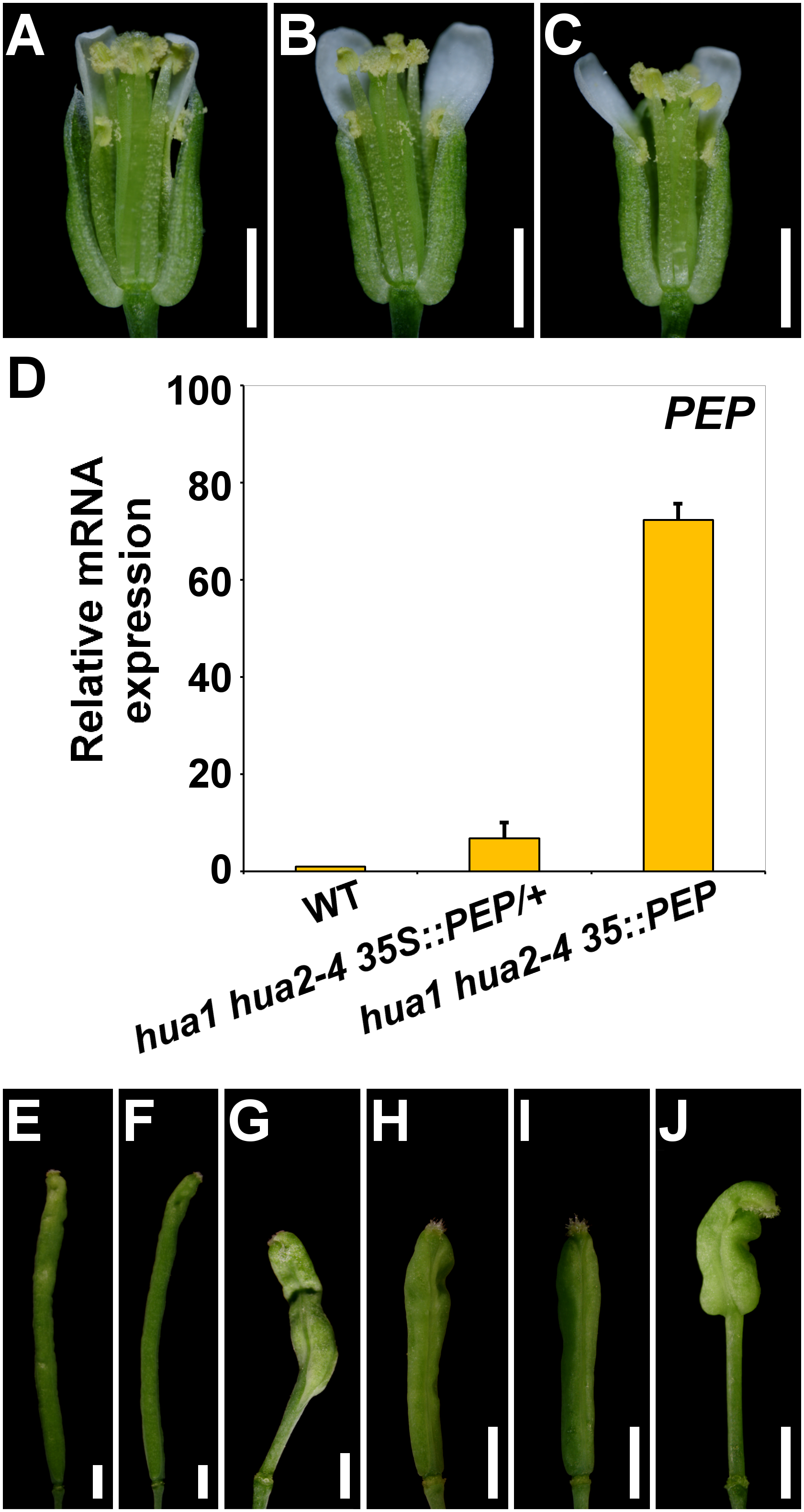

Supplement: S10 Fig — A) Double homozygous hen4 35S::PEP flower. B) Double homozygous hua1 35S::PEP flower. C) Double homozygous hua2 35S::PEP flower. D) PEP mRNA levels were monitored by qPCR in the wild type and hua1 hua2-4 plants hemizygous (hua1 hua2-4 35S::PEP/+) or homozygous (hua1 hua2-4 35S::PEP) for the 35S::PEP construct, respectively. Transgenic plants produced much higher levels of PEP transcript than the wild type. We used the hua2-4 allele [73] (SALK_032281) containing caulimoviral 35S promoter sequences, potentially able to trigger silencing of overexpressor lines driven by the same promoter element [Daxinger et al., 2008 in S2 Table]. E-G) The phenotype of hua1 hua2-4 gynoecia (E) was not modified in hua1 hua2-4 35SPEP/+ plants (F), but appeared dramatically enhanced in triple homozygous hua1 hua2-4 35S::PEP plants (G). H-J) Similar results were obtained with the hua2-7 allele [74] (SAIL_314_A08) lacking 35S sequences in its T-DNA. H) hua1 hua2-7 gynoecium. I) hua1 hua2-7 hua1 hua2-7 35S::PEP/+ gynoecium. J) hua1 hua2-7 35S::PEP gynoecium. In panels (A-C) some outer organs were manually removed to visualize inner organs. Scale bars: 1 mm. Error bars, SD. (TIFF) [file pgen.1004983.s010.tiff]

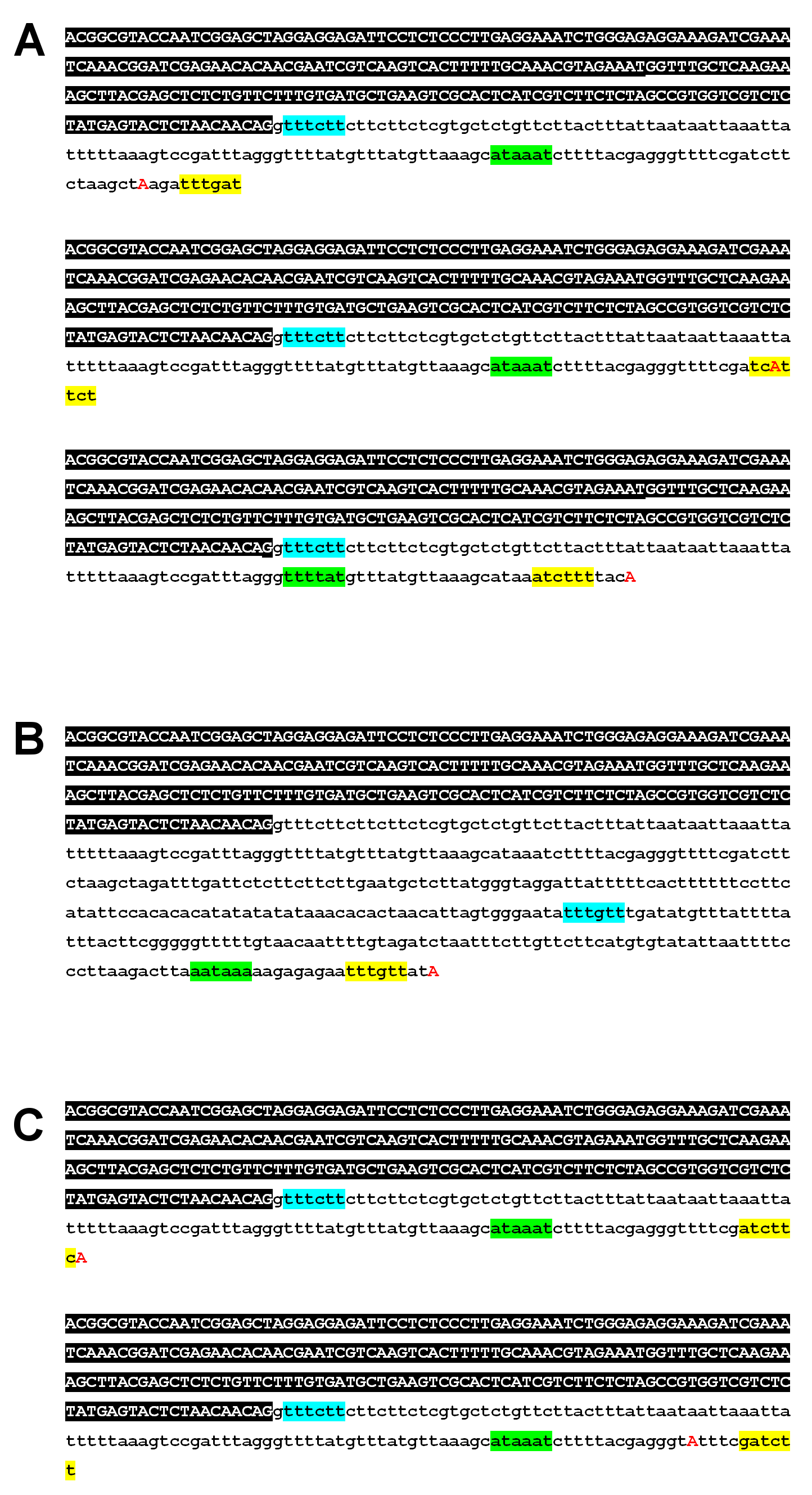

Supplement: S11 Fig — DNA sequence corresponding to exon 2 appears as white upper-case letters boxed in black. Intron 2 sequence is shown as lower-case black letters. Cleavage site is indicated (A in red). Putative cis-elements associated to cleavage and polyadenylation are depicted boxed in blue (FUE), green (NUE) and yellow (CE), respectively [63]. A) Some prematurely processed transcripts identified in the hua1 hua2 pep/+ mutant background. B) An aberrantly processed transcript identified in the hua1 hua2 35S::PEP mutant background. The most abundant version of FUE in plants (UUUGUU boxed in blue), and a canonical AAUAAA sequence element for NUE (green) are indicated [63]. C) Prematurely terminated transcripts identified in the flk hua1 hua2 mutant background. (TIFF) [file pgen.1004983.s011.tiff]

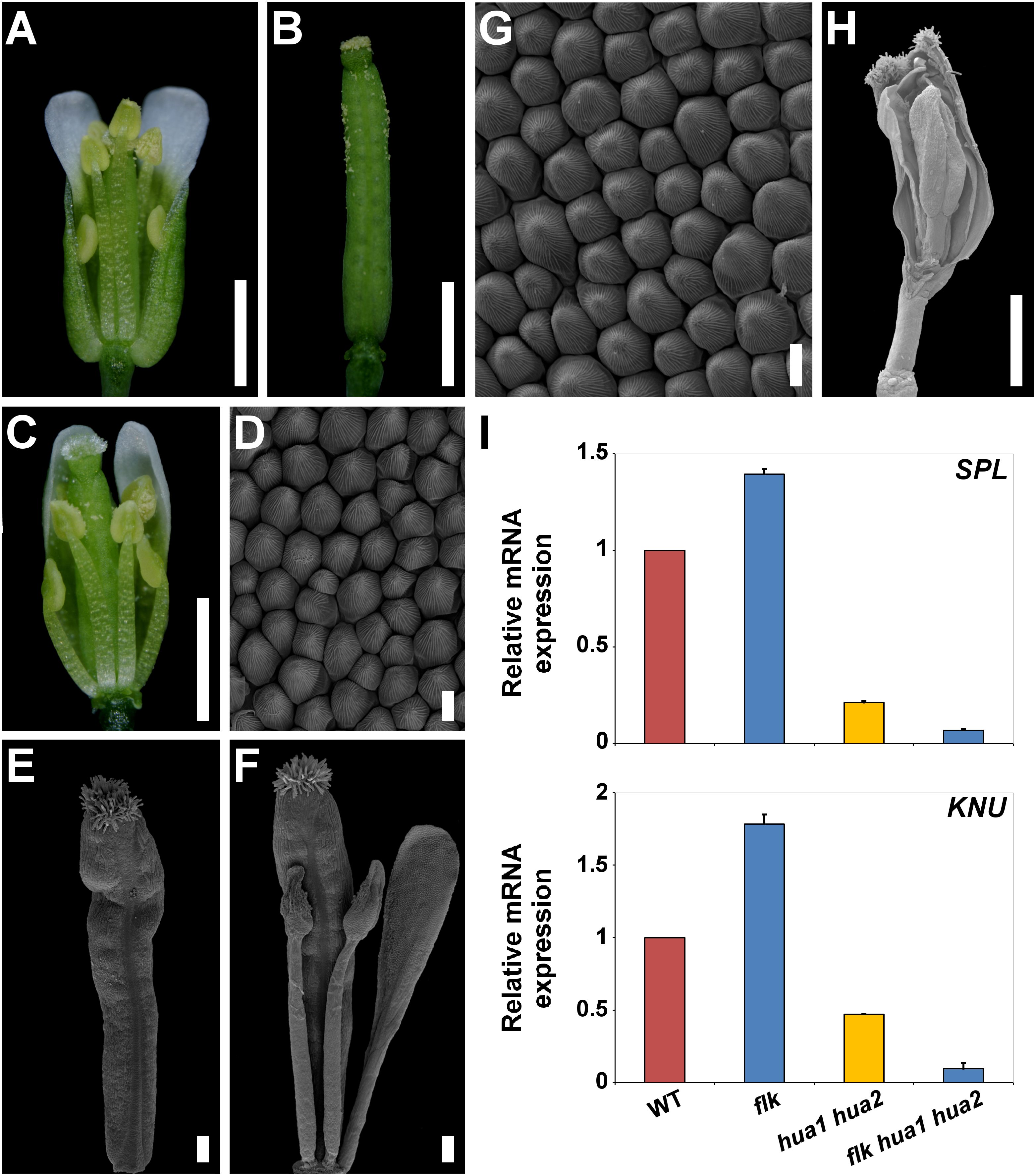

Supplement: S12 Fig — Floral phenotypes and RNA expression data. A) Wild-type looking flk hen4 double mutant flower. Some outer organs were manually removed to show normal looking stamens. B) Young developing flk hen4 fruit. C) Wild-type looking flk hua1 double mutant flower. Some outer organs were manually removed to show normal looking stamens. D-F) SEM images of flk pep hua1/+ flower organs. D) Corrugated pistil. E) A flower after removing most organs around the pistil, except three third whorl organs with obvious petaloid traits. F) Close-up view of the adaxial surface of a petaloid area in a third whorl organ in (E) showing conically shaped cells. G, H) SEM images of flk hua1 hua2 flower organs. G) Petaloid conically shaped cells on the adaxial surface of a third whorl organ. H) A flower in which a fourth whorl organ was manually removed to uncover additional flowers inside. I) Relative mRNA expression of SPL and KNU in the wild type (WT) and diverse mutant backgrounds, monitored by qPCR. Error bars, SD. Scale bars: 1 mm (A, B, C, H), 200 μm (E, F), 10 μm (D, G). (TIFF) [file pgen.1004983.s012.tiff]

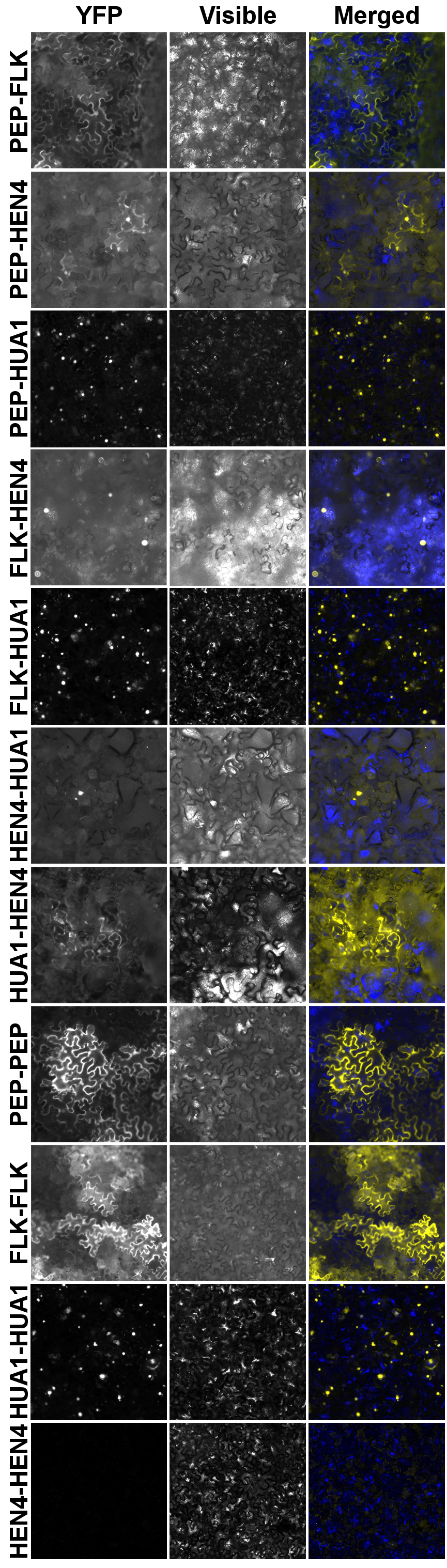

Supplement: S13 Fig — Visualization of YFP reconstitution (yellow fluorescence) in Nicotiana benthamiana leaf cells agroinfiltrated with plasmids encoding fusion proteins. The first 5 interactions show the reciprocal assays of those depicted in Fig. 6 (the first protein fused to the YFPct, and the second protein to the YFPnt). The interaction between HEN4 and HUA1 [28] was confirmed in both directions and used as a positive control. The ability of PEP, FLK and HUA1 to homodimerize was also verified. HEN4 was unable to homodimerize, thus providing a negative control. As further negative controls, Nicotiana leaves were co-infiltrated with the corresponding recombinant YFPct construct and the empty YFPnt version. The reciprocal assays were also performed. No signal was detected in any case. In merged visible+YFP fluorescence pictures, blue background has been used to increase the contrast. (TIFF) [file pgen.1004983.s013.tiff]

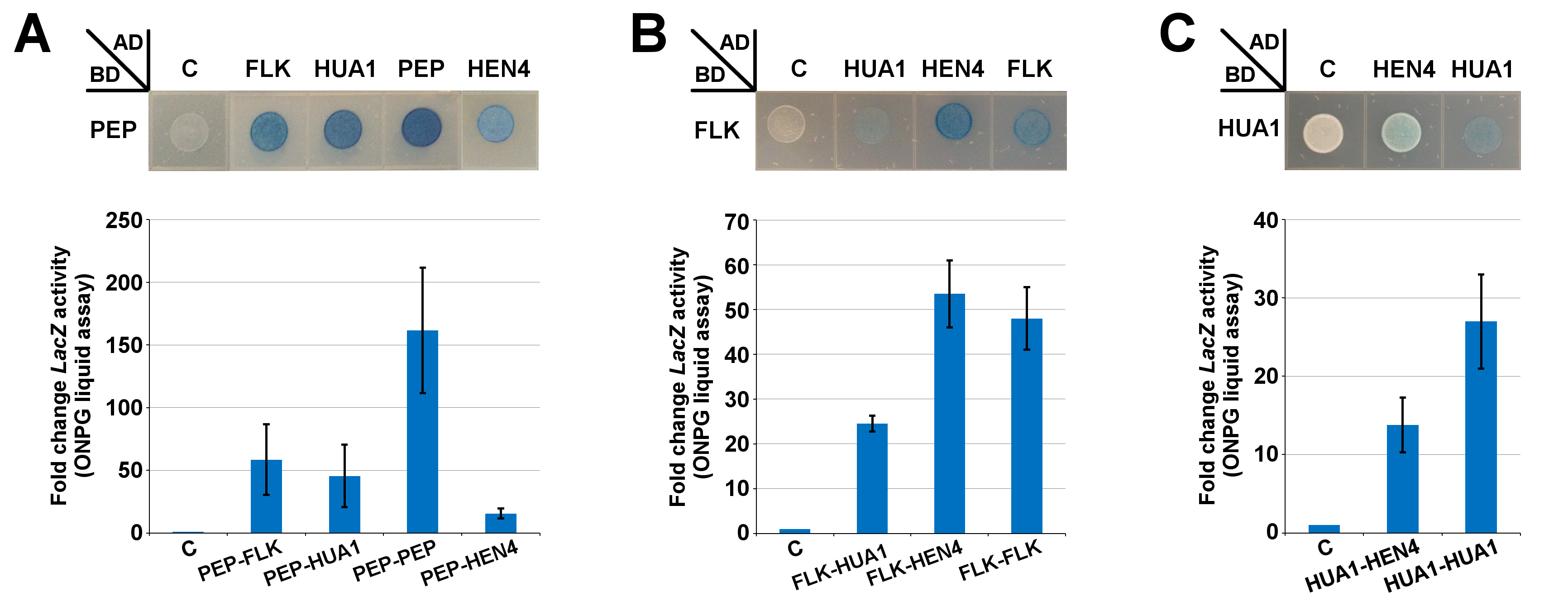

Supplement: S14 Fig — Protein interactions for PEP (A), FLK (B), and HUA1 (C). Induced yeast X-Gal plate assays are shown on the top of each panel. Y2H liquid assays using the reagent ONPG are shown below. Error bars indicate standard deviation (SD). (TIFF) [file pgen.1004983.s014.tiff]
