# Supplementary material for: K-homology Nuclear Ribonucleoproteins Regulate Floral Organ Identity and Determinacy in Arabidopsis
Source: PLoS Genet. 2015 Feb 6;11(2):e1004983. doi: 10.1371/journal.pgen.1004983 (PMC4450054; doi:10.1371/journal.pgen.1004983)
Supplement: S1 Table — For each genotype approximately 75 flowers from 5 different plants were examined. Flowers were collected from top, bottom and intermediate zones of each plant. Petaloid transformations of stamens were arbitrarily classified as mild (totally or partially transformed lateral stamens), intermediate (transformed lateral stamens and partially converted medial stamens), and strong (all indistinguishable from second whorl petals). In hua1 hua2, pistils with four valves were extremely scarce whereas in the other two genotypes were easily found. (DOCX) [file pgen.1004983.s015.docx]

**Table S1. Loss and overexpression of *PEP* enhance the mutant phenotypes of *hua1 hua2* flowers.**

**GENOTYPES**

| **PHENOTYPEs** | | ***hua1 hua2*** | ***hua1 hua2 pep/+*** | ***hua1 hua2 35S::PEP*** |
| --- | --- | --- | --- | --- |
| **Petaloid organs in the 3rd whorl** | Mild  Medium  Strong  Total | 62%  36%  2%  100% | 6%  82%  12%  100% | 14%  32%  54%  100% |
| **Apically unfused gynoecia** | | 54% | 97% | 100% |
| **New organs in additional whorls** | | 0% | 24% | 64% |
| **Supernumerary valves** | | 4% | 32% | 56% |
| **Long gynophore** | | 0% | 35% | 72% |
