# Supplementary material for: K-homology Nuclear Ribonucleoproteins Regulate Floral Organ Identity and Determinacy in Arabidopsis
Source: PLoS Genet. 2015 Feb 6;11(2):e1004983. doi: 10.1371/journal.pgen.1004983 (PMC4450054; doi:10.1371/journal.pgen.1004983)
Supplement: S2 Table — (DOCX) [file pgen.1004983.s016.docx]

**Table S2. Oligonucleotides, genotyping and additional references.**

| **Purpose** | **Name** | **Oligonucleotide sequence (5’-3’)** | | **Gene** |
| --- | --- | --- | --- | --- |
| qPCR | ACT2-f | CTCTTAACCGTAAAGCTAACAG | | *ACT* |
|  | ACT2-r | AGTGAGAATCTTCATGAGTGAG | | *ACT* |
|  | AGE9 | GAAGTGGTCAAAAGAAACATAG | | *AG* |
|  | AGI c | CCTACCCATAAGAGCATTCAAGAAG | | *AG* intron 2 |
|  | AG QPCR F | AGCAATCACGGCGTACCAATC | | *AG* / *AG* intron 2 |
|  | FUP1-37 | TCTGCTTTTGTGACACAGGTATCT | | *PEP* |
|  | FUP1-38 | CCTTGTGATTGATGATGAACTCTTGA | | *PEP* |
|  | KNU QPCR F | AAAACTCGATCATCAAGCAAACGC | | *KNU* |
|  | KNU QPCR R | GTAGATCCTTGAAATGGGTTTTGG | | *KNU* |
|  | NZZ qF | AGTCGGAGACACGTCATCAGTA | | *SPL/NZZ* |
|  | NZZ qR | GAAGAAGATACTGATCGTAGCCGT | | *SPL/NZZ* |
|  | OTC F | TGAAGGGACAAAGGTTGTGTATGTT | | *OTC* |
|  | OTC R | CGCAGACAAAGTGGAATGGA | | *OTC* |
| 3’ RACE | AGI a | CGGATCGAGAACACAACGAATCG | |  |
|  | AGI b | GGTTTGCTCAAGAAAGCTTACGAGC | |  |
| **Purpose** | **Name** | **Oligonucleotide sequence (5’-3’)** | **Modifications** | |
| BiFC | bifc FLK-f | CTATTTACAATTGTCGACATGGCTGAAGCTGAAGATC | | DNA assembly tail (SalI) |
|  | bifc FLK-r | TTTCGAACCCGGGGTACCTAACCGTAGCCTGAGCTG | | DNA assembly tail (KpnI) |
|  | bifc HEN4-f | CTATTTACAATTGTCGACATGGAGCGAAATAGCGTTAAATTTCATGC | | DNA assembly tail (SalI) |
|  | bifc HEN4-r | TTTCGAACCCGGGGTACCGTTTAGGTTGTATTTTTTGGACAATGAGG | | DNA assembly tail (KpnI) |
|  | bifc HUA1-f | CTATTTACAATTGTCGACATGGCACATCGTCAATTGTATAG | | DNA assembly tail (SalI) |
|  | bifc HUA1-r | TTTCGAACCCGGGGTACCTTGAGTAGTGTCGGTGTTGGTTGC | | DNA assembly tail (KpnI) |
|  | bifc PEP-f | CTATTTACAATTGTCGAC TGGCCGCCGTCGCAGATTCCGTTG | | DNA assembly tail (SalI) |
|  | bifc PEP-r | TTTCGAACCCGGGGTACCAAGATTATAACTGCTGTAGCCACC | | DNA assembly tail (KpnI) |
|  |  |  | |  |
| Y2H | pGILDA-PEP-f | AACGGCGACTGGCTGGAATTCATGGCCGCCGTCGCAGATTCCG | | DNA assembly tail (EcoRI) |
|  | pGILDA-PEP-r | TTGGCTGCAGGTCGACTCGAGTCAAAGATTATAACTGCTGTAG | | DNA assembly tail (XhoI) |
|  | pB42AD-FLK-f | GATTATGCCTCTCCCGAATTCATGGCTGAAGCTGAAGATCAGC | | DNA assembly tail (EcoRI) |
|  | pB42AD-FLK-r | AGAAGTCCAAAGCTTCTCGAGTCAGTAACCGTAGCCTGAGCTG | | DNA assembly tail (XhoI) |
|  | pB42AD-HUA1-f | GATTATGCCTCTCCCGAATTCATGGCACATCGTCAATTGTATAG | | DNA assembly tail (EcoRI) |
|  | pB42AD-HUA1-r | AGAAGTCCAAAGCTTCTCGAGTCATTGAGTAGTGTCGGTG | | DNA assembly tail (XhoI) |
|  | pB42AD-PEP-f | GATTATGCCTCTCCCGAATTCATGGCCGCCGTCGCAGATTCCG | | DNA assembly tail (EcoRI) |
|  | pB42AD-PEP-r | AGAAGTCCAAAGCTTCTCGAGTCAAAGATTATAACTGCTGTAG | | DNA assembly tail (XhoI) |
|  | pGILDA-FLK-f | AACGGCGACTGGCTGGAATTCATGGCTGAAGCTGAAGATCAGC | | DNA assembly tail (EcoRI) |
|  | pGILDA-FLK-r | TTGGCTGCAGGTCGACTCGAGTCAGTAACCGTAGCCTGAGCTG | | DNA assembly tail (XhoI) |
|  | pGILDA-HUA1-f | AACGGCGACTGGCTGGAATTCATGGCACATCGTCAATTGTATAG | | DNA assembly tail (EcoRI) |
|  | pGILDA-HUA1-r | TTGGCTGCAGGTCGACTCGAGTCATTGAGTAGTGTCGGTG | | DNA assembly tail (XhoI) |
|  | pB42AD-HEN4-f | GATTATGCCTCTCCCGAATTCATGGAGCGAAATAGCGTTA | | DNA assembly tail (EcoRI) |
|  | pB42AD-HEN4-r | AGAAGTCCAAAGCTTCTCGAGCTAGTTTAGGTTGTATTTTTTGG | | DNA assembly tail (XhoI) |
| **Purpose** | **Name** | **Oligonucleotide sequence (5’-3’)** | | **PCR product size in base pairs** |
| *hua2-7* genotyping | hua2-4r | CAATATGATCTGTTCCATCCAC | | 983 wild-type  650 mutant |
|  | hua2-7 2F | CTCGCCAAAGTCAAAGGCT | |  |
|  | LBTM3 | TAGCATCTGAATTTCATAACCAATCTCGATACAC | |  |
| *35S::PEP* genotyping | FUP1-37 | TCTGCTTTTGTGACACAGGTATCT | | 244 |
|  | FUP1-38 | CCTTGTGATTGATGATGAACTCTTGA | |  |

The rest of genotyping procedures were as previously reported:

*flk-2* (Ripoll *et al.*, 2009) [46]

*ful-1* (Ferrándiz *et al.*, 2000) [57]

*hen4-2* (Cheng *et al.*, 2003) [28]

*hua1-1* (Western *et al.*, 2002)

*hua2-1* (Western *et al.*, 2002)

*hua2-4* (Ripoll *et al.*, 2009) [73]

*pep-4* (Ripoll *et al.*, 2006) [44]

*KNU::GUS*, *gAP1::GFP* and *gAG::GFP* are resistant to hygromycin

*ful-1*, *pep-4*, and *35S::PEP* are resistant to kanamycin

**Supplementary references**

Daxinger L, Hunter B, Sheikh M, Jauvion V, Gasciolli V, et al. (2008) Unexpected silencing effects from T-DNA tags in *Arabidopsis*. Trends Plant Sci 13: 4-6.

Western TL, Cheng Y, Li J, Chen X (2002) *HUA ENHANCER2*, a putative DExH-box RNA helicase, maintains homeotic B and C gene expression in *Arabidopsis*. Development 129**:** 1569-1581.
